# Targeting barrel field spiny stellate cells using a vesicular monoaminergic transporter 2-Cre mouse line

**DOI:** 10.1038/s41598-021-82649-8

**Published:** 2021-02-05

**Authors:** Fabio B. Freitag, Aikeremu Ahemaiti, Hannah M. Weman, Katharina Ambroz, Malin C. Lagerström

**Affiliations:** Department of Neuroscience, Uppsala University, 751 24 Uppsala, Sweden

**Keywords:** Neural circuits, Somatosensory system, Transporters in the nervous system

## Abstract

Rodent primary somatosensory cortex (S1) is organized in defined layers, where layer IV serves as the main target for thalamocortical projections. Serotoninergic signaling is important for the organization of thalamocortical projections and consequently proper barrel field development in rodents, and the vesicular monoamine transporter 2 (VMAT2) can be detected locally in layer IV S1 cortical neurons in mice as old as P10, but the identity of the Vmat2-expressing neurons is unknown. We here show that *Vmat2* mRNA and also Vmat2-Cre recombinase are still expressed in adult mice in a sub-population of the S1 cortical neurons in the barrel field. The Vmat2-Cre cells showed a homogenous intrinsically bursting firing pattern determined by whole-cell patch-clamp, localized radial densely spinous basal dendritic trees and almost exclusively lack of apical dendrite, indicative of layer IV spiny stellate cells. Single cell mRNA sequencing analysis showed that S1 cortical Vmat2-Cre*;tdTomato* cells express the layer IV marker *Rorb* and mainly cluster with layer IV neurons, and RNAscope analysis revealed that adult Vmat2-Cre neurons express *Vmat2* and vesicular glutamate transporter 1 (*Vglut1*) and *Vglut2* mRNA to a high extent. In conclusion, our analysis shows that cortical *Vmat2* expression is mainly confined to layer IV neurons with morphological, electrophysiological and transcriptional characteristics indicative of spiny stellate cells.

## Introduction

In mice, the somatosensory (S1) barrel cortex layer IV represents a major target area for thalamocortical projections from the ventroposteromedial nucleus (VPM) and holds an intrinsic map of the rodents vibrissae^1^, which enables transmission of the discriminate component of sensory stimuli from that area of the animal. Layer IV consists of both inhibitory and excitatory neurons where the latter are mainly comprised of spiny stellate and star pyramidal cells^2,3^. Spiny stellate cells are characterized by large NMDAR-mediated excitatory postsynaptic potentials (EPSPs)^4^, radially symmetric and asymmetric dendrites^5^ that are confined within a single barrel^6^, and the lack of an apical dendrite^7^.

Altered glutamatergic signaling in the thalamocortical circuit during development results in mice with malfunctioning sensory function as seen in plasticity-related gene-1 knockout mice^8^. Similarly, adult mice with reduced tactile input from the whiskers during the neonatal period display defects in sensory tests and altered spiny stellate cell morphology, hinting at a role for stellate cells in tactile processing^9^. Spiny stellate cells communicate with layer VI pyramidal cells^10,11^, which project back to thalamic structures^12^, thus enabling a continuous feedback system. Additionally, layers II/III, IV and V neurons are also postsynaptic targets of spiny neurons^11^. Thus, although this cell type has processes confined to its home column and dendrites located within the same barrel, axonal branches can be spread through all cortical layers, suggesting the functional importance of spiny stellate cells in cortical processing.

The vesicular monoamine transport 2 (VMAT2) is responsible for transporting monoamines such as serotonin, dopamine and noradrenaline into vesicles in the pre-synapse^13,14^. In the adult mouse brain, the expression of *Vmat2* mRNA is restricted to monoaminergic neurons but the gene is also transiently expressed in non-aminergic cells during development^15^. *Vmat2* mRNA can, for instance, be found in the hippocampus, the medial and lateral ventro-posterior thalamic nuclei (VPM and VPL), and in layer IV of the somatosensory cortex^16^. A controlled level of serotonin is needed for a proper formation of the barrel fields^17^, which may represent one explanation for the transient expression of *Vmat2*^18^.

Here, by screening the expression of *Vmat2* in the mouse nervous system through the use of the Vmat2-Cre mouse line, we observed that cre recombinase is still expressed in adult S1 cortical neurons. Interestingly, this expression was shown to be mainly confined to layer IV. By targeting this population by either crossing to the *tdTomato* reporter mice or administration of viral vectors, performing whole-cell patch-clamp, neuron reconstruction, single cell mRNA sequencing, immunohistochemistry and RNAscope analysis, we conclude that this population is composed of mainly S1 layer IV spiny stellate cells. Thus, we show that the Vmat2-Cre line can be used as a transgenic tool to study intrinsic properties, connectivity and function of layer IV spiny stellate cells in the adult mouse, contributing to a better understanding of how somatosensory stimuli are cortically processed and modulated.

## Results

### Vmat2-Cre*;tdTomato* expression can be identified in several somatosensory-associated areas in the mouse nervous system

**Figure 1:**
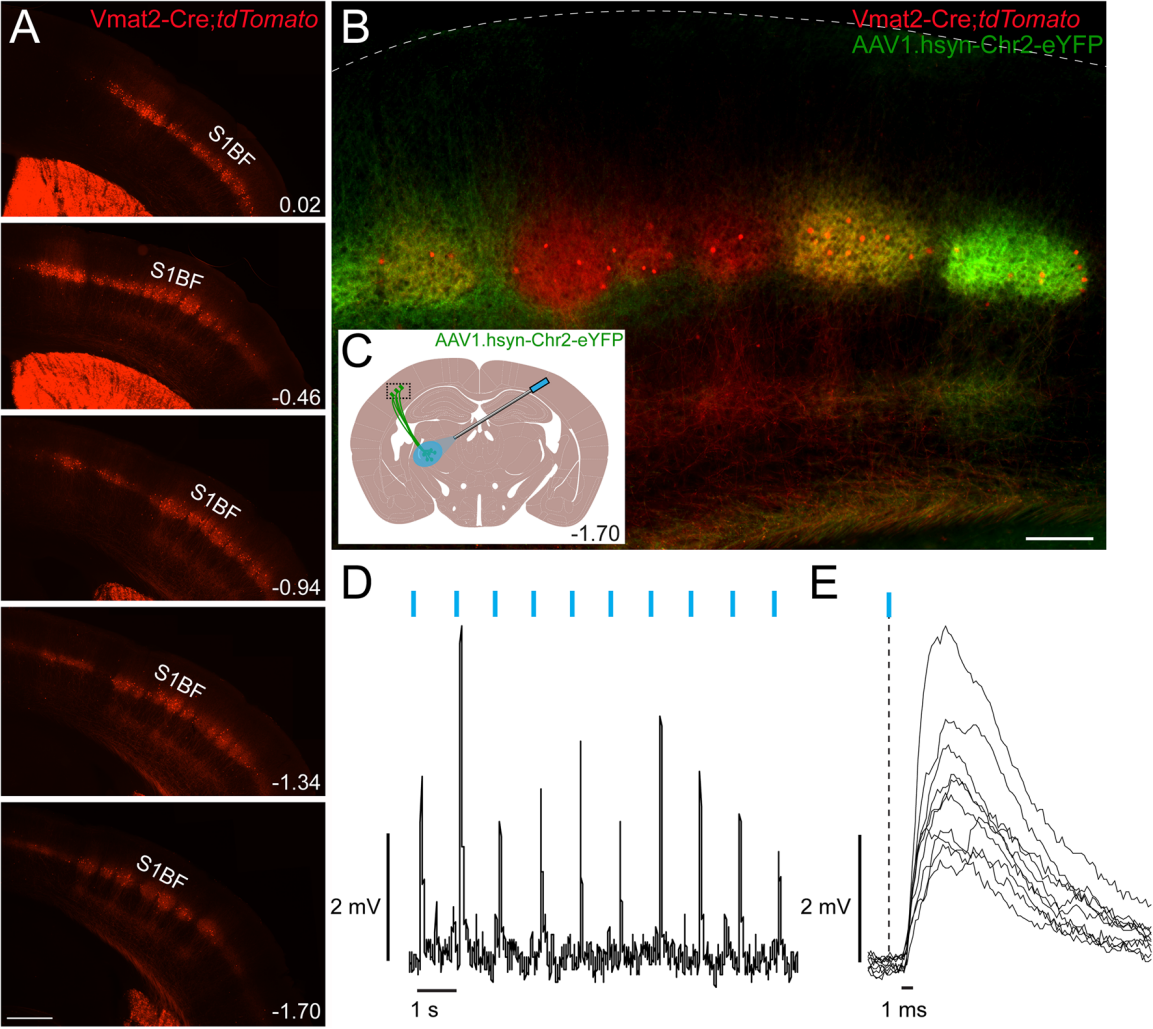
Vmat2-Cre;*tdTomato* expressing neurons in cortex layer IV are contacted by thalamocortical projections. (**A**) Vmat2-Cre;*tdTomato* expression in cortex is mainly confined to layer IV and spreads rostro-caudally (n = 3 mice). Scale bar 500 µm. The images are composites to enable a high resolution. The images were generated using Image J (Image J 1.53e), https://imagej.nih.gov/ij/. (**B**) Vmat2-Cre;*tdTomato* neurons are surrounded by thalamo-cortical projections revealed by *yfp* expression (green) from AAV1.hsyn-ChR2.eYFP virus injected into VPL/VPM thalamic nucleus. Scale bar 150 µm. The image is a composite to enable a high resolution. The image was generated using Fiji (ImageJ 1.52f.), https://imagej.net/Welcome. (**C**) Schematic drawing that displays the direction of the ChR2.eYFP expressing thalamocortical neurons innervating the Vmat2-Cre active neurons in the barrel cortex, shown in B, and the experimental setup. The anatomical figure is adopted from Paxinos and Franklin’s mouse brain atlas^38^. (**D**,**E**) Current clamp recording from a Vmat2-Cre;*tdTomato* S1BF cortical neuron. The figure (**D**) shows excitatory postsynaptic potentials (EPSPs) evoked by blue light pulses (473 nm) at 1 Hz (indicated by blue lines above EPSPs). Additionally, the superimposed EPSPs (**E**) show relatively consistent latency (the black vertical dashed line indicates the time point of the light stimulation), indicating monosynaptic connections from thalamo-cortical projections to recorded neurons. Images (**D**,**E**) were generated using WinWCP, Version 5.5.5, http://spider.science.strath.ac.uk/sipbs/software_ses.htm. S1BF, primary somatosensory cortex barrel field.

First, we set out to study the overall expression of Vmat2-Cre in somatosensory-associated areas of the central nervous system and dorsal root ganglia of the mouse using the reporter line *tdTomato*. Weak Vmat2-Cre activity could be detected in dorsal root ganglia and in the dorsal spinal cord, whereas the trigeminal nuclei in the brainstem were devoid of Vmat2-Cre activity (Figure S1A,B). A high density of Vmat2-Cre;*tdTomato* expressing cells could be detected in several areas of thalamus, including the VPL/VPM (Figure S1C), and somatosensory barrel cortex S1 layer IV (Fig. 1A, Figure S1C).

### Layer IV Vmat2-Cre neurons are contacted by thalamocortical projections

The barrel field layer IV is a major thalamo-cortical input layer in the primary somatosensory cortex. Thus, to validate the anatomical position of *tdTomato*-marked Vmat2-Cre cortical neurons, we injected AAV1.hsyn-ChR2-eYFP virus in the VPL/VPM area of the thalamus. By doing so, thalamo-cortical projections could be seen forming clear barrels in cortical layer IV and surrounding Vmat2-Cre;*tdTomato* neurons (Fig. 1B,C). Whole-cell patch-clamped Vmat2-Cre;*tdTomato* neurons (n = 12 from 3 mice) were current clamp recorded while pulses of light were delivered to the brain slices. As shown in Fig. 1D, excitatory postsynaptic potentials (EPSPs) were observed for each pulse of light at 1 Hz without failure (Fig. 1D). Additionally, the induced EPSPs (Fig. 1E) also showed relatively consistent latency (4.0 ± 0.3 ms, n = 12), confirming that the recorded Vmat2-Cre;*tdTomato* neurons receive monosynaptic inputs from thalamic VPL/VPM projections.

### Vmat2-Cre;*tdTomato* cells show intrinsically bursting firing pattern and similar morphological properties

**Figure 2:**
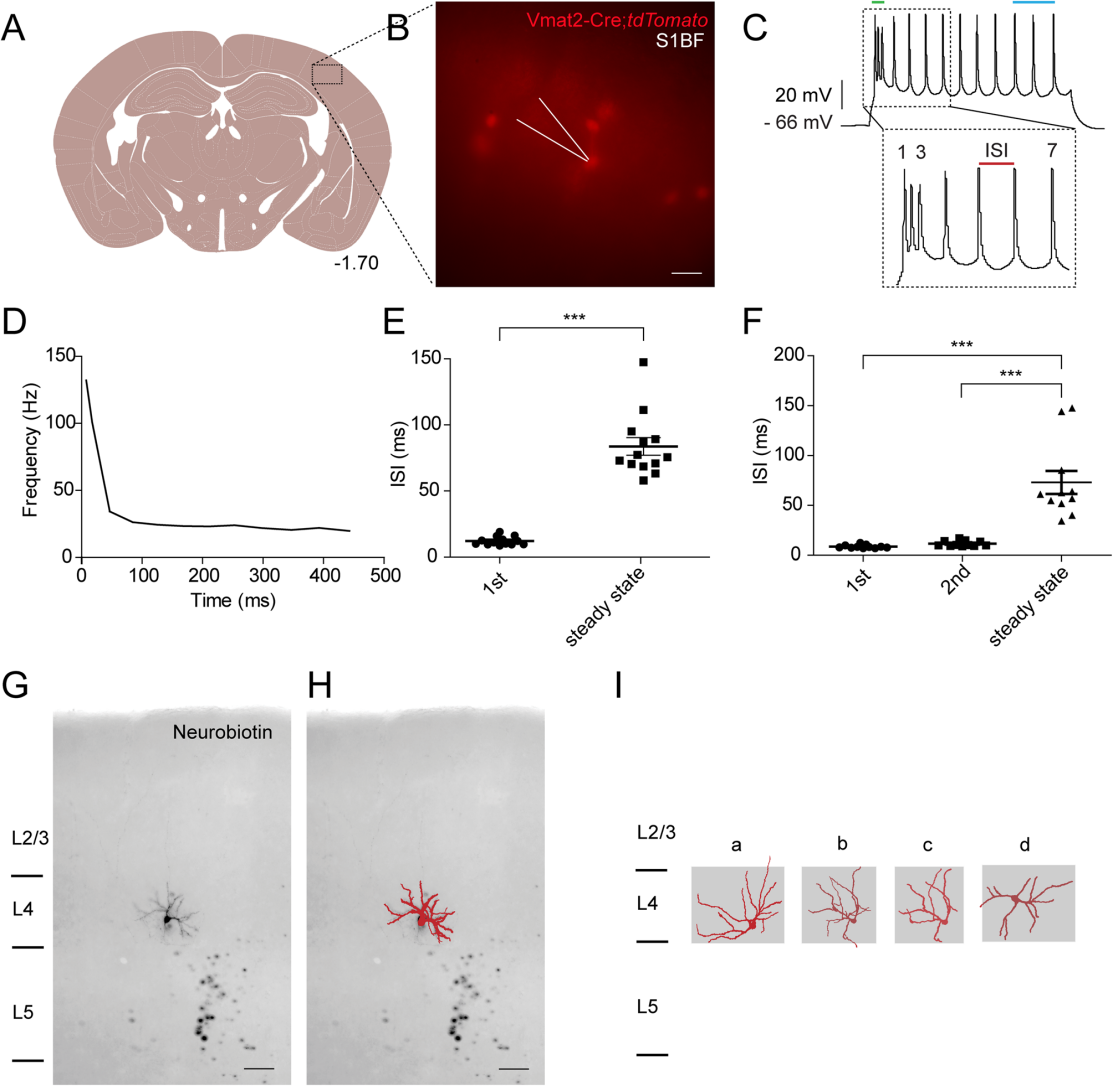
Morphology and electrophysiological properties of the Vmat2-Cre;*tdTomato* cells. (**A**) Whole cell patch clamp recordings were performed in coronal slices preserving the barrel field cortex. The anatomical figure is adopted from Paxinos and Franklin’s mouse brain atlas^38^. (**B**) Vmat2-Cre;*tdTomato* expressing cells in somatosensory cortical layer IV. The white arrowhead represents the pipette for patch-clamp recording. Scale bar 25 µm. The image was generated using Image J (Image J 1.51 k), https://imagej.nih.gov/ij/. (**C**) A representative trace of the induced action potential, which demonstrated an initial bursting property. The initial bursting phase is marked with a green line and the steady state phase (the last three spikes) is marked with a blue line. I-S-I is the time interval between two spikes. The image was generated using WinWCP, Version 5.5.5, http://spider.science.strath.ac.uk/sipbs/software_ses.htm. (**D**) Frequency plot of the recorded action potential in (**B**). (**E**,**F**) Comparison of I-S-I of recorded action potentials (***p < 0.0001, one-way ANOVA with Bonferroni’s post hoc test) between initial bursting phase and the steady state phase. The initial bursting phases of all the recorded action potentials (n = 24) consist of either two spikes (**E**, n = 13) or three spikes (**F**, n = 11). Images (**D–F**) were generated using GraphPad Prism version 5.0.1 for Windows, GraphPad Software, San Diego, California USA, www.graphpad.com. (**G**) A Neurobiotin-stained cell (10 × magnification). Cortical layers are marked on the left. The figure is shown in inverted gray scale for a better visualization. (**H**) The overlay of reconstructed dendrites (red) from the Vmat2-Cre neuron in (**G**). Scale bar 40 µm. (**I**) Representative examples of reconstructed Neurobiotin-stained Vmat2-Cre;*tdTomato* cells showing either asymmetrical (a, b and c) or symmetrical (d) dendritic trees. The scale bar in (**F**) also applies to (**I**). Images (**G–I**) were generated using Simple Neurite Tracer (https://imagej.net/SNT)^54,55^ in Fiji, https://imagej.net/Welcome.

Whole-cell patch-clamp recording was used to investigate the electrophysiological properties of layer IV Vmat2-Cre;*tdTomato* cells. Resembling the firing pattern of excitatory neurons in layer IV^19^, all the patched Vmat2-Cre;*tdTomato* cells (n = 24 from 13 mice) with an average resting membrane potential of − 68.9 ± 1.6 mV (Fig. 2A,B) showed an intrinsically bursting firing pattern upon electrical stimulation with an average rheobase of 42.9 ± 4.7 pA (Fig. 2C). Intrinsic bursting can be detected by the significantly higher firing frequency in the initial phase of the stimulated action potential compared to the steady state phase (the last three action potential) (Fig. 2D) or equally by the significant increase of I-S-I after the initial spikes (Fig. 2E,F). I-S-I analysis revealed that 54% of the recorded cells (13 out of 24) demonstrated two initial burst spikes, where the I-S-I increased significantly after the first interval (Fig. 2E, ***p < 0.0001) while the rest 46% had three initial burst spikes (Fig. 2F, ***p < 0.0001).

To analyze the morphology of Vmat2-Cre neurons, Vmat2-Cre;*tdTomato* cells were filled with Neurobiotin (Fig. 2G) or adult Vmat2-Cre neurons were studied through Cre-dependent virus-mediated mCherry expression (Figure S2A–D). The Neurobiotin filled Vmat2-Cre neurons showed a lack of apical dendrites and dendritic trees remaining within a barrel in layer IV of the somatosensory cortex (Fig. 2G–I). We chose to analyze the dendritic morphology using two different methods where the viral-based technique was used to evaluate active Cre expression during adulthood through Cre-dependent viruses, while the neuron reconstruction was performed on Vmat2-Cre;*tdTomato* cells filled with Neurobiotin. The viral-based analysis also identified two Vmat2-Cre neurons with an apical dendrite, representing 0.5 ± 0.5% of the Vmat2-Cre population per section (2 out of 312 neurons, 10 sections from five mice), which indicates that a few adult Vmat2-Cre neurons could represent pyramidal neurons (Figure S2C,D). The Neurobiotin analysis identified Vmat2-Cre;*tdTomato* neurons with either restricted asymmetrical (n = 7/10) or symmetrical (n = 3/10) dendritic trees. Thus, viral vectors confirm that the Cre recombinase enzyme is still expressed during the Vmat2-Cre adulthood, and the expression seems mainly restricted to barrel field spiny stellate cells, as indicated by neuron reconstruction.

### The Vmat2-Cre*;tdTomato* population is molecularly homogenous and adult Vmat2-Cre cells show prevalent expression of *Vglut1* and *Vglut2*

**Figure 3:**
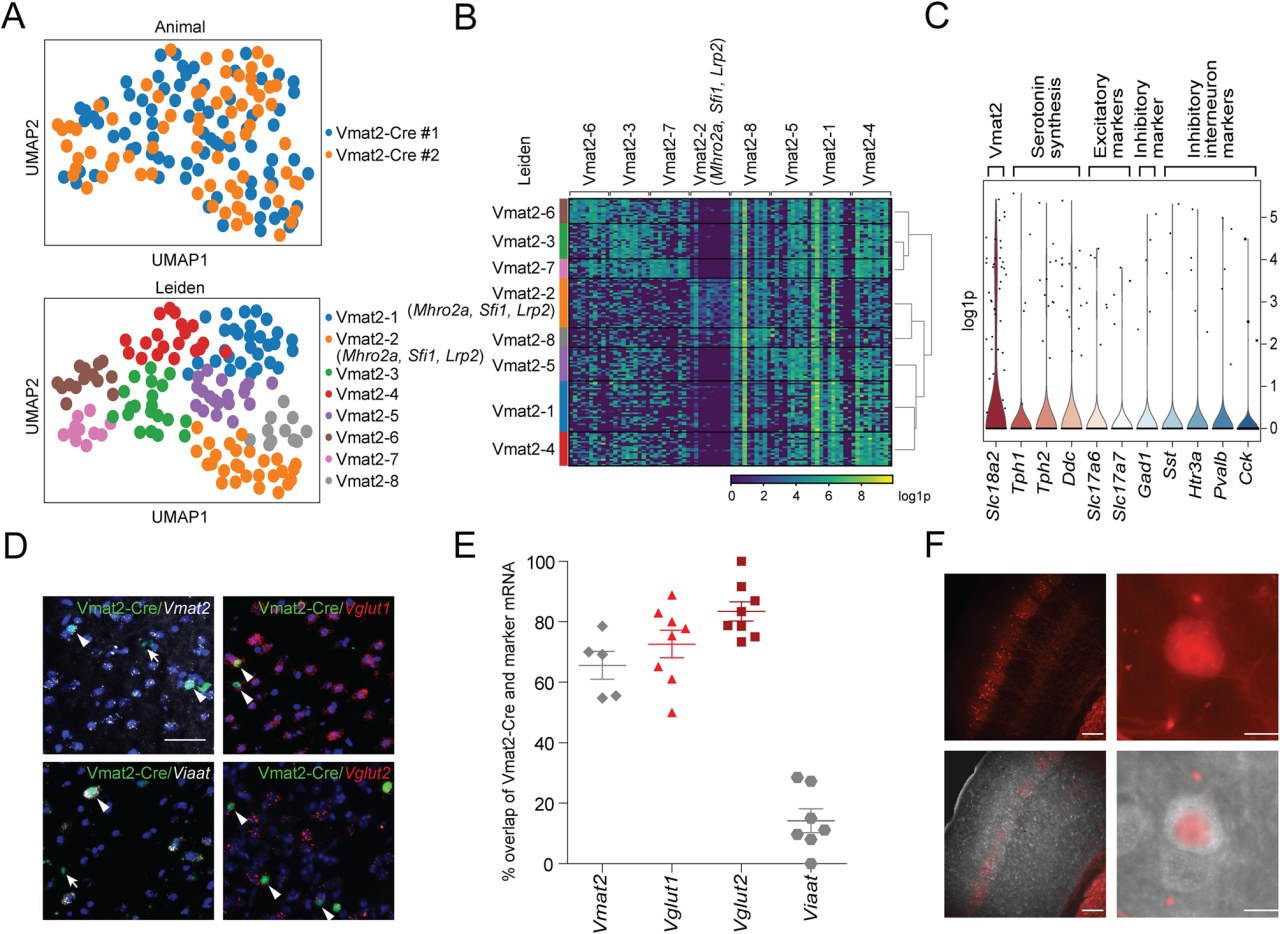
Single-cell mRNA sequencing reveals homogeneity of the Vmat2-Cre;*tdTomato* population. (**A**) Somatosensory cortical Vmat2-Cre;*tdTomato* cells from two animals were single-cell mRNA sequenced using Smart-Seq2. Leiden clustering shows that there are 8 clusters of Vmat2-Cre cells, but only one of these clusters (Vmat2-2) shows a significant expression for top differential expressed (DE) marker genes (FDR < 0.05). (**B**) Heatmap of expression of DE genes for individual Vmat2-Cre cells according to assigned Leiden cluster. (**C**) Violin plot of the expression of target neurotransmitter marker genes per cell in the Vmat2-Cre:*tdTomato* population (gene considered expressed if log1p ≥ 0.25). Images (**A–C**) were generated using SCANPY, https://scanpy.readthedocs.io/en/stable/. (**D**) RNAscope detection of neurotransmitter markers *Vmat2* (grey, top left), *Vglut1* (red, top right), *Viaat* (grey, bottom left) and *Vglut2* (red, bottom right) in SB1F Vmat2-Cre.HTB neurons (green) (scale bar 100 µm). Arrowhead indicates Vmat2-Cre.HTB neuron expressing targeted gene and arrow indicates Vmat2-Cre.HTB neuron not expressing the targeted gene. The images were generated using Fiji (ImageJ 1.52f.), https://imagej.net/Welcome. (**E**) Scatter plot showing the percentage of adult Vmat2-Cre.HTB neurons that express the targeted gene ± SEM. The image was generated using GraphPad Prism version 5.0.1 for Windows, GraphPad Software, San Diego, California USA, www.graphpad.com. (**F**) Immunostaining was performed to detect GABAergic Vmat2-cre*;tdTomato* cells in the barrel field cortex (left). Scale bar 200 µm. A Vmat2-cre*;tdTomato* cell expressing GABA (right). Scale bar 10 µm. The images were generated Image J (Image J 1.53e), https://imagej.nih.gov/ij/.

To investigate the molecular profile of the Vmat2-Cre cells, we first performed single cell sequencing on Vmat2-Cre;*tdTomato* cells to investigate the expression profile of the population as a whole, followed by an RNAscope analysis on the adult Vmat2-Cre neurons visualized through a Cre-dependent viral-based technique. The single-cell mRNA sequencing was performed on 178 somatosensory cortical Vmat2-Cre;*tdTomato* neurons using Smart-Seq2^20^. A total of 24,582 genes could be detected, and after basic preprocessing, 18,430 genes were used for the analysis of the remaining 158 neurons (exclusion of low quality cells and low-prevalent genes). The normalized sequenced cells shared topology similarity among the two animals (Figure S3A, Fig. 3A), indicating an equivalent Vmat2-Cre;*tdTomato* representation from both animals. To investigate if the Vmat2-Cre:*tdTomato* consists of sub clusters that could be defined by differentially expressed (DE) genes, the dataset was clustered. The clustering of the neurons resulted in 8 detected populations, however, only one of the clusters showed significant expression for top DE genes (FDR < 0.05) for glial markers such as *Fcer1g*, *Ciqc* and *Ctss* (mousebrain.org) (Figure S3A,B), and therefore, the 7 cells belonging to this cluster were excluded. The exclusion resulted in 151 cells that were re-clustered into 8 clusters (Fig. 3A). Again, even though these clusters were defined, only one of them showed significant differential gene expression (FDR < 0.05, cluster Vmat2-2, top significant expression for *Mroh2a*, *Lrp2*, *Sf11*) (Fig. 3B, Figure S4, Table S1), indicating that the Vmat2-Cre*;tdTomato* population is generally molecularly homogenous on cluster level. Therefore, instead of studying the expression of target genes for the individual clusters, the expression was examined for the whole Vmat2-Cre*;tdTomato* population. The Vmat2-Cre*;tdTomato* population was found to express *Slc18a2* (*Vmat2*), and this gene was prevalent in 25.2% of the adult cells (gene prevalent if log1p ≥ 0.25). Moreover, genes that catalyze the synthesis of serotonin; *Tph1* and *Tph2* (tryptophan-5-hydroxylase), were found in 3.3% and 7.9%, respectively, whereas *Ddc* (aromatic L-amino acid decarboxylase) was detected in 9.9% (Fig. 3C). Markers for excitatory neurons, including the vesicular glutamate transporter (Vglut) genes; *Slc17a6* (Vglut2) and *Slc17a7* (Vglut1) were found in a smaller subset of the Vmat2-Cre*;tdTomato* population (*Slc17a6*: 4.6%, *Slc17a7*: 3.3%) whereas the inhibitory markers *Slc32a1* (Viaat) was not detected in the dataset and *Gad1* was only detected in 3.3% (Fig. 3C). Moreover, low-to-zero expression and prevalence for inhibitory interneuron markers, such as *Sst* (somatostatin), *Htr3a* (5-hydroxytryptamine receptor 3a), *Pvalb* (parvalbumin), *Calb2* (calretinin), *Npy* (neuropeptide Y), *Vip* (vasoactive intestinal polypeptide), and *Cck* (cholecystokinin)^21–24^ were found (Fig. 3C, *Sst*: 2%, *Htr3a:* 3.3%, *Pvalb*: 3.3%, *Calb2*: 0%, *Npy*: 0%, *Vip*: 0%, *Cck*: 2% (genes with 0 expression are not displayed in the figure)) (for a receptor expression analysis, please see Figure S5). The single cell sequencing analysis thus revealed that the Vmat2-Cre;*tdTomato* cells, in general, displayed a low prevalence of investigated neurotransmitter marker mRNA (except *Vmat2*) and to further investigate the neurotransmitter phenotype of Vmat2-Cre cells, and in particular Vmat2-Cre cells with active Cre expression in adults, the expression of several neurotransmitter markers were analyzed using RNAscope. Vmat2-Cre expression in cortex layer IV was visualized through a Cre-dependent viral-based technique where AAVDJ.EF1a-DIO-HTB was injected in adult mice (n = 2). The analysis revealed that 65.6 ± 4.1% (69/108 cells) of the Vmat2-Cre.HTB neurons expressed *Vmat2*, 72.6 ± 4.3% (178/237 cells) *Vglut1*, 83.5 ± 2.2% (123/149 cells) *Vglut2* and 14.2 ± 3.6% (18/125 cells) *Viaat* (Fig. 3D,E), indicating that the adult Vmat2-Cre population is mainly an excitatory population. Immunostaining against the inhibitory neurotransmitter GABA further confirmed these findings as only 2.6 ± 0.4% of Vmat2-Cre;*tdTomato* cells expressed GABA (Fig. 3F).

### The Vmat2-Cre population expresses the cortical layer IV marker *Rorb*

**Figure 4:**
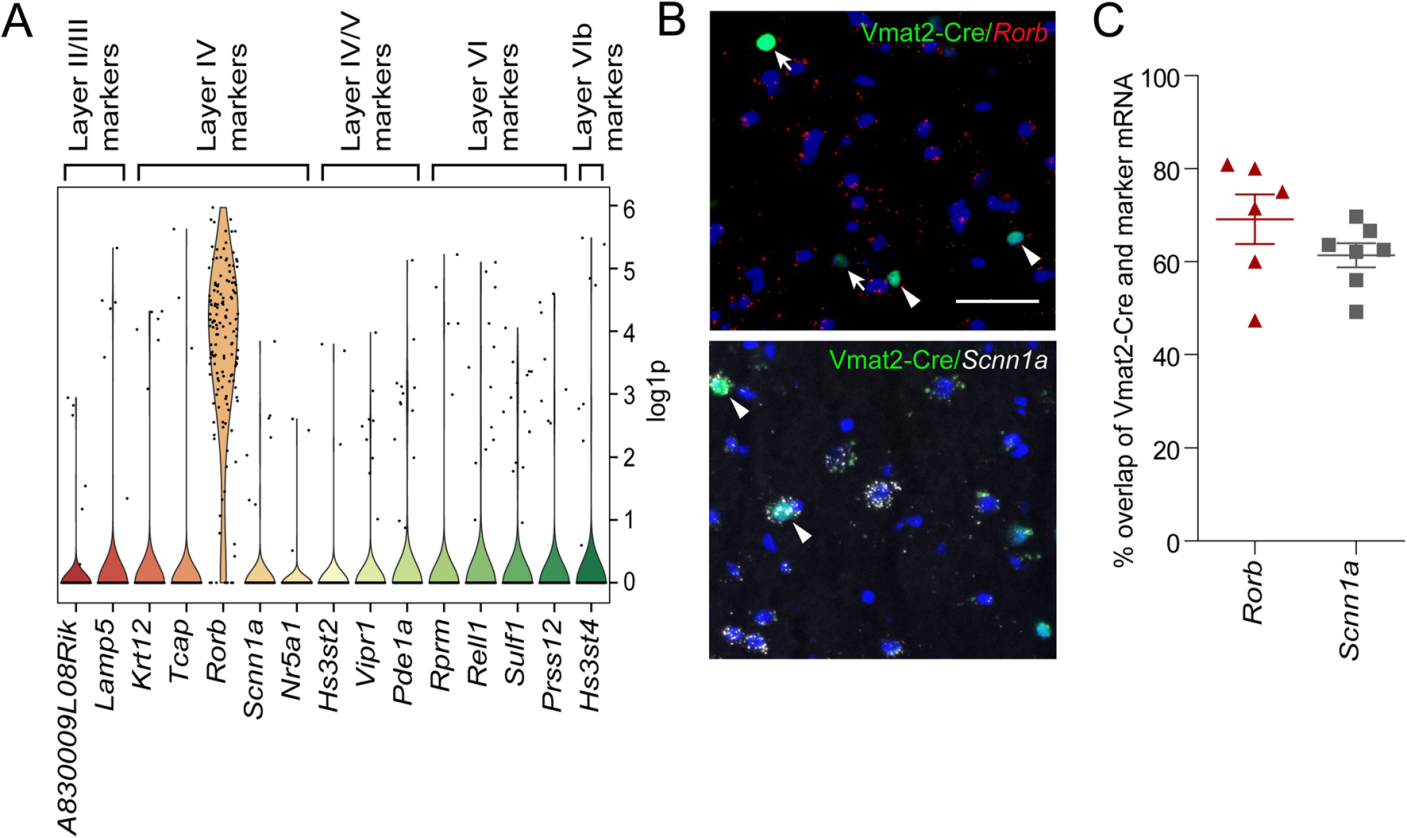
The IV layer marker *Rorb* is expressed by the majority of Vmat2-Cre neurons. (**A**) Violin plot of the expression of Zeisel et al. cortical layer markers in the Vmat2-Cre;*tdTomato* population (gene considered expressed if log1p ≥ 0.25). The image was generated using SCANPY, https://scanpy.readthedocs.io/en/stable/. (**B**) RNAscope detection of the SB1F Vmat2-Cre.HTB (green) expression of layer IV markers *Rorb* (red) and *Scnn1a* (grey) (scale bar 100 µm). Arrowhead indicates Vmat2-Cre.HTB neuron expressing targeted gene and arrow indicates Vmat2-Cre.HTB neuron not expressing the targeted gene. The images were generated using Fiji (ImageJ 1.52f.), https://imagej.net/Welcome. (**C**) Scatter plot showing the percentage of adult Vmat2-Cre.HTB neurons that express the targeted layer IV marker gene ± SEM. The image was generated using GraphPad Prism version 5.0.1 for Windows, GraphPad Software, San Diego, California USA, www.graphpad.com.

To molecularly examine the location of the Vmat2-Cre neurons, the expression of cortical layer markers^25^ was analyzed in the Vmat2-Cre;*tdTomato* single cell dataset. The expression of layer II/III markers *A830009L08Rik*, *Lamp5* and *Gm12371* was low (*A830009L08Rik*, *Lamp5* expressed by 4%, *Gm12371* not detected), whereas layer IV marker *Rorb* (the annotated name for *RORβ*) was the most prominently expressed of the target genes (expressed by 92.7%). Other prominent layer IV markers *Krt12*, *Tcap*, *Scnn1a*, *Nr5a1* and *Tbr2*^26^ were lowly detected (*Krt12*: 4%, *Tcap*: 2%, *Scnn1a*: 4.6%, *Nr5a1*: 2%, *Tbr2*: 0%) (Fig. 4A). Low prevalence of the layer V markers *Hs3st2*, *Vipr1* and *Pde1a* was detected (*Hs3st2*: 5.3%, *Vipr1*: 6%, *Pde1a*: 8.6%). Lastly, for the layer VI markers, *Rprm*, *Rell1*, *Sulf1*, *Prss12* and layer VIb markers *Hst3st4, Cplx3* and *Nxph3* were also lowly detected in the Vmat2-Cre population (*Rprm*: 3.3%, *Rell1*: 7.9%, *Sulf1*: 9.3%, *Prss12*: 5.3%, *Hst3st4*: 5.3%, *Cplx3* and *Nxph3* not detected) (Fig. 4A). This targeted gene expression analysis indicates that the Vmat2-Cre population is mainly located in a subset of layer IV neurons of the somatosensory cortex predominately marked by *Rorb*.

To validate the layer marker expressional analysis, RNAscope was performed targeting the expression of *Rorb* and *Scnn1a* (Fig. 4B) in Vmat2-Cre.HTB cortical neurons, where both of these markers were found to be expressed by more than half of the Vmat2-Cre.HTB neurons (*Rorb*: 69.1 ± 4.9%, 77/115 cells expressed *Rorb*; *Scnn1a*: 61.4 ± 2.4%, 121/205 cells expressed *Scnn1a*) (Fig. 4C). In conclusion, the RNAscope analysis showed that the layer 4 markers *Rorb* and *Scnn1a* are expressed in the adult Vmat2-Cre expressing population.

**Figure 5:**
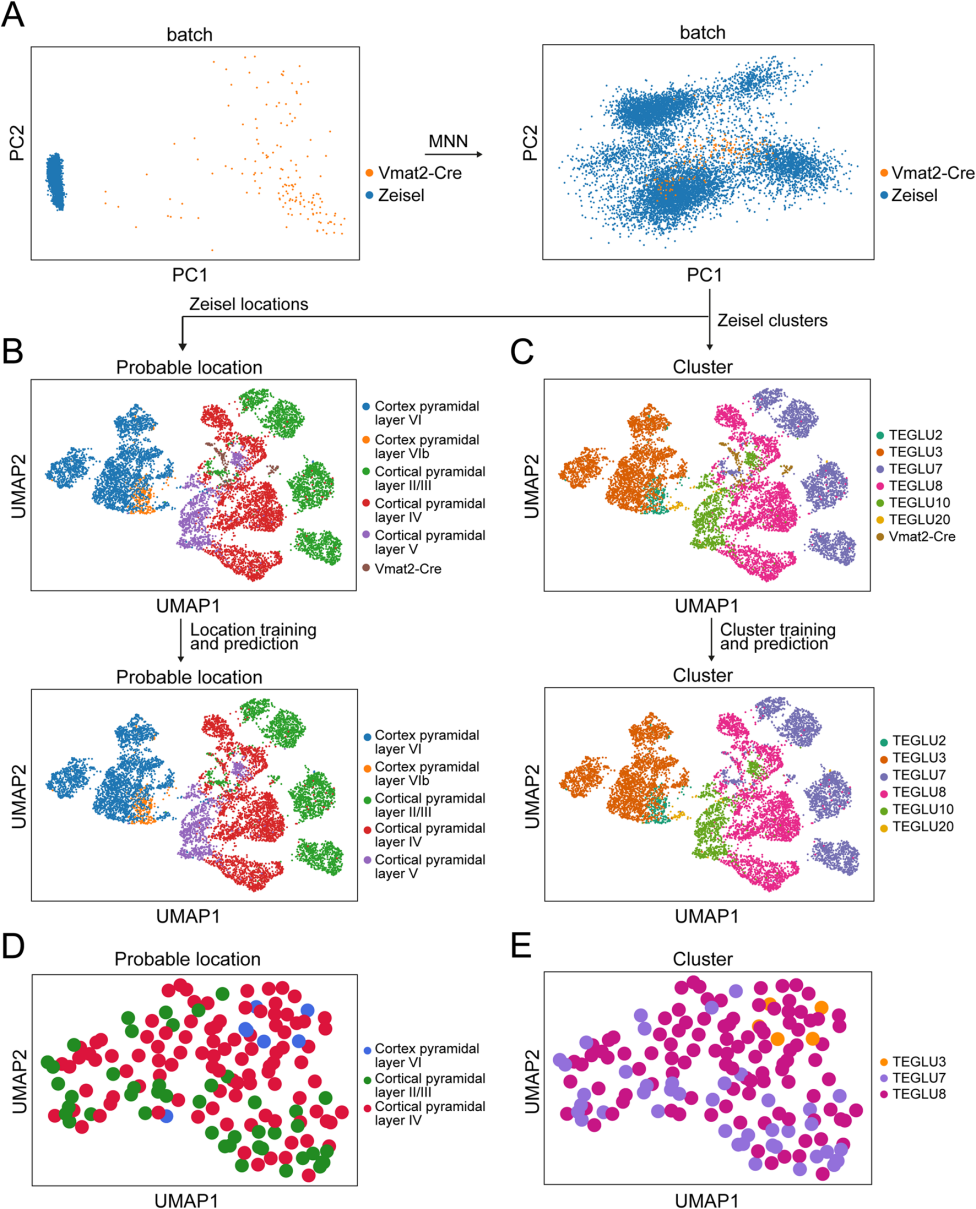
The Vmat2-Cre;*tdTomato* population is mainly located in layer IV. (**A**) The Vmat2-Cre single cell sequencing data was merged with the Zeisel et al. telencephalon projecting cortical dataset to molecularly predict the location and cluster of the Vmat2-Cre;*tdTomato* population. Mutual nearest neighbors (MNN) was used to correct for gene expression caused by batch effects. (**B**) A stochastic gradient descent (SGD) classifier was trained on the Zeisel et al. dataset to predict the possible location of the Vmat2-Cre cells. (**C**) The Zeisel et al. cluster annotations of the Vmat2-Cre neurons were predicted using a SGD Classifier. (**D–E**) Replotting of the Vmat2-Cre population with Zeisel et al. annotated locations (**D**) and clusters (**E**). The images (**A–E**) were generated using SCANPY, https://scanpy.readthedocs.io/en/stable/.

To further examine the location and molecular cell types of the Vmat2-Cre population in a greater context, our Vmat2-Cre dataset was concatenated with the cortical sub-dataset of single-cell mRNA sequencing data from the telencephalon projecting excitatory neuron dataset published by Zeisel et al. (2018). After isolation of the target cortical cells and basic preprocessing, the data of 17,304 genes expressed by 9369 cells from the Zeisel dataset was concatenated and aligned with our Vmat2-Cre dataset using mutual nearest neighbors (MNN) (Fig. 5A), resulting in a total of 9520 cells and 14,425 shared expression genes. The annotated clusters and probable locations defined by Zeisel et al. were thereafter predicted for the Vmat2-Cre cells (Fig. 5B,C). From the labeled location predictions, 45 Vmat2-Cre cells belonged to cortical pyramidal layer II/III (29.8%), 99 to layer IV (65.6%) and 7 to layer VI (4.6%) (Fig. 5B). From the cluster assignment, 5 Vmat2-cells belonged to TEGLU3 (3.3%), 42 to TEGLU7 (27.8%) and 104 to TEGLU8 (68.9%) (Fig. 5C). The TEGLU3 cluster is likely located in layer VI, TEGLU7 in layer II/III and TEGLU8 in layer IV (mousebrain.org), indicating that the probable location and cluster assignments of the Vmat2-Cre population were consistent. This consistency could also be indicated by mapping the Zeisel et al. probable location and cluster annotations to the original Vmat2-Cre dataset, where the location-cluster annotations overlapped (Fig. 5D,E). Collectively, both the marker gene analysis of Vmat2-Cre, the mapping of the population to a previously published cortical dataset and in situ hybridization show that the Vmat2-Cre population is primarily located in cortical layer IV.

## Discussion

Here, we show that a subpopulation of somatosensory cortex layer IV neurons expresses *Vmat2* mRNA and Cre in adult Vmat2-Cre mice. The expression is confined to a population of mainly excitatory *Vglut1* and/or *Vglut2* expressing neurons with dendritic processes located within single barrels and almost exclusively lacking apical dendrites, and displaying an intrinsically bursting firing pattern upon stimulation, indicative of spiny stellate cells. The single cell sequencing, immunohistochemistry and RNAscope analysis also showed that Vmat2-Cre is active in a small subset of inhibitory interneurons. The single cell mRNA sequencing analysis further showed that the adult Vmat2-Cre cells mainly cluster with layer IV cortical neurons and display the layer IV marker *Rorb*, which was confirmed by the RNAscope analysis.

In the mouse brain, expression of *Vmat2* mRNA can still be found at P10 in layer IV of the somatosensory cortex^16^. *Vmat2* null mice show small areas of densely packed cells at P5 instead of clear barrels and in monoamine oxidase A null mice the granular neurons in layer IV instead form a continuous layer^17^. Also, increased levels of serotonin during development impairs barrel field formation^27^. Our analysis reveals that *Vmat2* is still found in a sub-population of layer IV barrel cortex neurons in the adult mouse. Low expression levels of genes needed for serotonin formation are found in Vmat2-Cre;*tdTomato* neurons, which may indicate that the synthesis and release of serotonin can occur in adult Vmat2-Cre neurons. Moreover, VMAT2 involvement in other monoamine pathways could also be considered. Additionally, unilateral vision loss in adult mice leads to increased expression of VMAT2 in the somatosensory barrel field^28^, indicating that VMAT2 may be needed to enable plasticity at adult stages after a sudden sensory loss.

Several studies have reported intrinsic electrophysiological properties of layer IV excitatory neurons^7,19,29^. Regular-spiking and intrinsically bursting spiking are consistently shown as the action potential signatures of excitatory pyramidal and spiny stellate cells in the thalamo-cortical recipient layer, with no clear distinction between spike profile and neuronal types^7,19^. We show that all Vmat2-Cre;*tdTomato* layer IV barrel field whole-cell patch-clamped neurons showed intrinsically bursting firing properties, pointing to a cortical spatially restricted population composed of excitatory neurons. By single-cell mRNA sequencing of Vmat2-Cre;*tdTomato* neurons, we could detect that the population is in general molecularly homogenous. However, as the expression of excitatory markers *Vglut1-2* was low and that inhibitory marker *Gad1* and inhibitory interneuron markers (*Sst*, *Htr3a*, *Pvalb* and *Cck*) also could be detected in low amounts, the single cell mRNA sequencing data was inconclusive to verify the excitatory properties of the Vmat2-Cre population indicated by the electrophysiological recordings. However, subsequent RNAscope analysis showed that a majority of the adult Vmat2-Cre cells expressed the excitatory markers *Vglut1* and/or *Vglut2*, which clarified that the Vmat2-Cre population is mainly excitatory. Both single-cell mRNA sequencing and RNAscope are sensitive methods for detecting gene expression and RNAscope analysis is commonly used to spatially verify the sequencing outcome, however, we could find inconsistencies in the prevalence of the targeted gene expression between these techniques. For instance, the prevalence of neurotransmitter marker genes was low and layer 4 marker *Rorb* was high in the sequencing data, whereas the neurotransmitter marker genes were more highly detected and *Rorb* expression lower in the RNAscope analysis. The explanation of these expressional differences could involve several factors, including the sequencing depth, method sensitivity and mRNA turn over^30^. Moreover, the sequencing was performed on developmentally marked Vmat2 neurons, whereas the RNAscope analysis was performed on adult Vmat2 neurons. The single cell sequencing and RNAscope analyses also identified a small inhibitory sub-population of Vmat2-Cre positive neurons. The identity of these neurons is however unknown as most markers for inhibitory neurons (*Sst*, *Htr3a*, *Pvalb*, *Calb2*, *Npy*, *Vip*, *Cck*) showed low-to-zero mRNA expression.

Our morphological analysis, which was based on cell filling and Cre- dependent virus mediated targeted fluorescent marking, shows that this population has dendritic trees mainly restricted to its home barrels and lacking apical spread, which are clear morphological evidence excluding the absolute majority of the neurons in this population as pyramidal neurons^31^. We did identify a few (0.5 ± 0.5% per section) adult Vmat2-Cre positive neurons with apical dendrites, which could indicate that the Vmat2-Cre population also contains a very limited number of pyramidal neurons. Thus, we conclude that in the Vmat2-Cre mouse line, the Cre enzyme is still expressed in adult mice and marks mostly cortical spiny stellate cells.

Spiny stellate cells have been shown to express *Rorβ* (RAR-related orphan receptor beta) mRNA^32^ and are together with pyramidal neurons, part of a population marked by the *Scnn1a* gene^31^. *Rorβ*^26,33^*, Scnn1a*^26^ and *Nr5a1*^26^ have indeed been used as markers to the cortical layer IV. However, evidence pointing to specific markers at the cell type level is still lacking. When analyzing the expression of the Zeisel et al. (2018) cortical layer markers and the Harris et al. (2014) layer IV markers, it was shown that *Rorb* was highly prevalent in the Vmat2-Cre*;tdTomato* population, whereas other layer IV markers such as *Scnn1a* and *Nr5a1* were less prevalent. When verifying the single cell sequencing expression of *Rorb* and *Scnn1a* using RNAscope for adult Vmat2-Cre neurons, expression was found in the majority of these neurons, molecularly indicating that the Vmat2-Cre population is located in layer IV. Indeed, when further validating the location and molecular profile of the Vmat2-Cre population by merging and aligning the Vmat2-Cre dataset with the Zeisel et al. (2018) excitatory cortical sub-dataset, we could see that the Vmat2-Cre neurons consist mainly of cortical layer 4 TEGLU8 neurons, located in cortical layer 4. Based on the histological findings, however, it would have been expected to find more Vmat2-Cre neurons mapped to the layer IV neurons. The sensitivity varies between the commercial single cell mRNA sequencing methods^34^ and data discrepancies may also arise from technical differences in dissociation protocols. The Zeisel et al. (2018) dataset was acquired with 10 × Genomics, whereas our Vmat2-Cre dataset was generated using Smart-Seq2, which may have affected the sequencing outcome. Even though computational methods for correcting for methodological differences exist^22,35–37^, no method can perfectly correct for the methodological differences, which may also be reflected by the lower probability scores (Figure S6D,E). Also, the Zeisel et al. dataset contained data from the whole cortex, and thus, the restricted Vmat2-Cre layer IV population in the barrel cortex may not have been captured to great extent. Conclusively, the histological findings, expression analysis of cortical layer markers and the mapping of the Vmat2-Cre dataset to the Zeisel et al. excitatory cortical dataset indicate that the Vmat2-Cre population is mainly located in cortical layer IV.

Here, we show that by using the mouse line Vmat2-Cre, barrel field spiny stellate cells can be targeted by either crossing with the *tdTomato* mouse line or through Cre-dependent viral vectors, as Cre is still present at adult ages. Future targeting and potentially manipulating the function of this cell type, and visualizing its activity in vivo through calcium imaging and optogenetics, can provide important insights and conclusions about how barrel field spiny stellate cells process specific somatosensory stimuli.

## Methods

### Animals

Animal procedures were approved by the local ethical committee in Uppsala (Uppsala djurförsöksetiska nämnd) and followed the Directive 2010/63/EU of the European Parliament and of the Council, The Swedish Animal Welfare Act (Djurskyddslagen: SFS 1988:534), The Swedish Animal Welfare Ordinance (Djurskyddsförordningen: SFS 1988:539), and the provisions regarding the use of animals for scientific purposes: DFS 2004:15 and SJVFS 2012:26. The Vmat2-Cre line (STOCK Tg(Slc18a2-cre)OZ14Gsat/Mmucd (MMRRC)) was used separately or crossed with the reporter line *tdTomato* (Gt(ROSA)26Sor^tm14(CAG-tdTomato)Hze^; Allen Brain Institute). The following primers were used to identify Cre and *tdTomato*, respectively; Cre 5′-acgagtgatgaggttcgcaaga-3′ (forward, mutant allele), 5′-accgacgatgaagcatgtttag-3′ (reverse, mutant allele) *tdTomato* 5′-tgttcctgtacggcatgg-3′ (forward, mutant allele), 5′-ggcattaaagcagcgtatcc-3′ (reverse, mutant allele), 5′-aagggagctgcgtggagta-3′ (forward, wild type allele), 5′-ccgaaaatctgtgggaagtc-3′ (reverse, wild-type allele). The Vmat2-Cre allele was kept heterozygous and both female and male mice were included in the analysis if not otherwise indicated. The study was carried out in compliance with the ARRIVE guidelines.

### Imaging of Vmat2-Cre;tdTomato expression

Adult (5 females and 1 male, 12–16 weeks old) Vmat2-Cre;*tdTomato* mice were perfused (see below) using PBS followed by 4% formaldehyde (FA). Whole brain, spinal cord and dorsal root ganglia tissue were dissected out. Brains and two spinal cords were kept in 4% FA overnight at 4 °C, then mounted in 4% agarose (VWR) and cut in 60 µm thick sections using a vibratome (Leica VT1200). After the FA incubation, one spinal cord with intact dorsal root ganglia tissue was placed in 15% sucrose (Sigma-Aldrich, USA) in 1 × PBS for 24 h, followed by 24 h incubation in 30% sucrose in 1 × PBS for 24 h for cryoprotection. The tissue was thereafter embedded in optimal cutting temperature (OCT) medium (Bio-Optica, Milan, Italy) and snap-frozen on dry ice in − 80 °C isopentane (Sigma-Aldrich, Germany). The tissues were cut into 18 µm sections using a cryostat (Leica Cryocut 1800) and collected onto Superfrost Plus (Thermo Scientific) slides.

The slices were subsequently visualized using an Olympus BX61WI fluorescence microscope (Olympus, Japan), where red fluorescence (tdTomato expression) was imaged and edited using ImageJ (ImageJ, USA) and Adobe Photoshop (to create composites) software. Atlases^38,39^ were used to determine the anatomical position of the tissue.

### Microinjection of viral vectors

To mark thalamocortical projections in the barrel field cortex, adult Vmat2-Cre;*tdTomato* mice (5 females, 10–11 weeks old) were injected with 500 nL of AAV1.hsyn.hChR2(H134R)-EYFP virus (abbreviated AAV1.hsyn-ChR2.eYFP in the result section, lot number v67891) from Addgene at a titer of 1.1 × 10^13^ cg/ml in the thalamic VPM area. The coordinates were B: -1.80; ML: 1.75; DV: 3.70. Alternatively, to mark cortical barrel field Vmat2-Cre cells in adult mice (2 males and 3 females, 11 weeks old), the same volume of AAV8.hsyn-DIO-mCherry (lot number v61605, titer: 2.20 × 10^13^ cg/ml) from Addgene, or AAVDJ.EF1a-DIO-HTB (lot date 2017, titer 1.83 × 10^11^ vg/ml) virus, produced by the Salk Institute GT3 (Gene Transfer, Targeting, and Therapeutics) core facility, was injected into S1 cortex of Vmat2-Cre mice (2 females, 10–23 weeks old). The coordinates used were B: 0.00; ML: 2.75; DV: 0.90.

### Tissue preparation for electrophysiology

Mice (7 males, 8 females; 12–52 weeks old,) were anesthetized with ~ 0.5 ml isoflurane (FORANE, Baxter, USA) for 1–2 min and injected i.p with 0.3 ml Ketamine (Ketalar, 10 mg/ml, Pfizer) and 0.3 ml Medetomidine (Domitor, 1 mg/ml, Orion Pharma). After about 4–5 min the effectiveness of the anesthesia was measured by paw pinch reflex. The mouse was fixed on a tissue paper with tape and the skin above the rib cage was removed. Part of the rib cage was removed carefully to expose the heart. The perfusion needle was inserted into the left ventral and a small cut was made to the right atrium. The mouse was perfused with cold NMDG-HEPES based cutting solution (concentration in mM: 93 N-methyl-d-glucamine, 2.50 KCl, 1.20 NaH_2_PO_4_, 30 NaHCO_3_, 20 HEPES, 25 Glucose, 5 sodium ascorbate, 2 Thiourea, 3 sodium pyruvate, 10 MgSO_4_∙7H_2_O, 0.5 CaCl_2_∙2H_2_O) for about 4 min with a peristaltic pump at a speed rate of 450 ml/hour. The brain was carefully removed from the skull and 300 µm thick coronal slices were made with a vibratome (Leica vt1200) in cold continuously oxygenated (95% O_2_ and 5% CO_2_) cutting solution (same as above) from B: − 0.10 to − 1.46 mm according to the mouse brain atlas stereotaxic coordinates^38^. The cut slices were then recovered in aCSF (concentration in mM: 126 NaCl, 2.5 KCl, 1.25 NaH_2_PO_4_, 26 NaHCO_3_, 10 glucose, 1.5 CaCl_2_, 1.5 MgCl2) at 36 °C for 1 h and at room temperature for a minimum of 30 min before they were placed in the recording chamber.

### Electrophysiology

The slices were then transferred to a recording chamber for electrophysiological analysis, according to a previously described procedure^40^, where Vmat2-Cre neurons were identified using a 60 × or 20 × water-immersion objective (LUMPlan FI, 0.90 numerical aperture (NA), Olympus) with the help of red fluorescence (tdTomato). In short, neurons were visualized on a Zyla sCMOS camera (Andor Technology Ltd) connected to a green (550 nm, CoolLED system) fluorescent LED light source. Patch electrodes (6–10 MΩ) were pulled from borosilicate glass capillaries (GC150F-10 Harvard Apparatus) with a flaming/brown micropipette puller (P-1000, Shutter Intrument, USA). The internal solution contained (in mM): 130 K-gluconate, 40 HEPES, 1.02 MgCl_2_, 2.17 MgATP, 0.34 NaGTP, with pH adjusted to 7.2 using 1 M KOH. Liquid junction potential was corrected before each patched neuron. Whole-cell patch-clamp signals were amplified with a MultiClamp 700B amplifier (Axon Instruments), digitalized at 20 kHz with Digidata 1440A (Molecular Devices), low pass filtered at 10 kHz, and acquired in WinWCP software (Dr. J. Dempster, University of Strathclyde, Glasgow, UK). Data analysis were done by Clampfit 10.3 (Molecular Devices, USA), Mini Analysis (Synaptosoft, USA) and Matlab (Mathworks). The action potential firing frequency was calculated by inverting the inter-spike interval (I-S-I), which is the time interval between two adjacent action potential peaks.

To verify the synaptic connection between barrel field cortical Vmat2-Cre;*tdTomato* neurons and thalamocortical afferent projections, the expression of channelrhodopsin 2 (ChR2) in the thalamic VPM area was first confirmed under 20 × water-immersion objective (LUMPlan FI, 0.90 numerical aperture (NA), Olympus) with the help of yellow fluorescence (eYFP). In the optogenetic experiment, the ChR2 expressing area was stimulated with blue light (473 nm) using (Mic-LED, Prizmatix, Israel) through an optic fiber placed about 5 mm from the area with an angle of 30 degrees at 1 Hz (10 ms pulse duration at 6 mV/mm^2^ of light power), while the responses from the *tdTomato* neurons were measured as EPSPs in the whole cell current clamp configuration. The latency was determined as the time delay from the starting point of a light pulse to the activation of a corresponded EPSP.

### Neurobiotin staining and imaging

Neurobiotin Tracer (Vector Laboratories, USA) was added into the intracellular solution (2–4 mg/ml) and delivered to the target cell during the patch-clamp recording. Depolarizing current pulses (0.5–1 nA, duration 150 ms) were injected into the cell through the recording electrode at 3 Hz for 5–10 min to improve the diffusion of Neurobiotin in the neurites. After the recording, the patch pipette was carefully retracted from the cell and removed from the slice. The slice was further perfused for at least 15 min to remove the excessive Neurobiotin in the tissue, which was then fixed in 4% paraformaldehyde (PFA, Histolab) overnight at 4 °C. The fixed slices were washed with phosphate-buffered saline (PBS, Fisher BioReagents) three times, 10 min each, and incubated in a staining solution containing streptavidin Alexa Flour 488 conjugate (Invitrogen), with a dilution ratio of 1:1000 in 0.3% Triton X-100 (Sigma) PBS solution for 4 h at room temperature on a rocker, and further incubated at 4 °C on a rocker for 72 h to obtain visualization of fine processes. The staining solution was removed after incubation and the slice was washed in 0.3% tritonX PBS solution two times, 20 min each and further washed once with PBS solution for 20 min. The slice was then mounted on a microscope slide with mounting media containing DAPI (ProLong Gold antifade reagent with DAPI, Invitrogen, USA). A coverslip was carefully placed on top of the slice and the edges were sealed with nail polish. The mounted slice was imaged using a Leica TCS SP8 confocal microscope with a water-immersion 25 × (HCX IRAPOL, 0.95 NA) objective. The imaged cells were traced and reconstructed using the Simple Neurite Tracer plug-in in the NIH ImageJ software (National Institutes of Health, Bethesda, Maryland). The presence of dendritic spines was used to distinguish between axons and dendrites and the analysis was focus on the dendritic processes to avoid incomplete reconstructions of potentially sliced axons.

### Tissue dissociation for single cell sequencing

Two adult (48–55 weeks old) female Vmat2-Cre;*tdTomato* and one *tdTomato* negative female (11 weeks old) were sedated in 4% isoflurane-containing box (FORANE, Baxter, USA) and injected i.p with 0.6 ml (1/1) mix of Ketamine (Ketalar, 10 mg/ml, Pfizer) and Medetomidine (Domitor, 1 mg/ml, Orion Pharma). The animals were thereafter perfused in ice-cold pre-oxygenated 1 × Dulbecco’s PBS (DPBS, Sigma-Aldrich). In the same solution, the forebrain was sectioned into 400 µm slices using a vibratome (Leica VT1200). Since many *tdTomato* cells could be detected in the striatum, the cortical *tdTomato* areas and the equivalent areas of the control tissue were carefully isolated using scissors. The cortical area from each animal was thereafter handled separately for dissociation. The dissociation was conducted using the Adult Brain Dissociation kit with minor modifications of the manufacturer’s instructions (Miltenyi Biotec, cat#: 130-107-667). The slices were placed in Eppendorf tubes containing preheated (37 °C) enzyme 1 (475 µl buffer Z, 25 µl enzyme P), where after 15 µl of preheated enzyme mix 2 (10 µl buffer Y, 5 µl enzyme A) was added. The tissues were dissociated for a total of 30 min at 37 °C; every 10 min, the tissues were triturated 10 times using Pasteur glass pipettes, where the diameter decreased for every trituration session. To inactivate the enzymes, the triturated cell suspensions were added to 5 ml ice-cold DPBS. A smartstrainer (40 µm, Corning) was presoaked in 3 ml DPBS and the cell suspension was thereafter added to the filter and an additional 2 ml DPBS was added to the filter to collect cells possibly remaining in the filter. The samples were centrifuged for 10 min at 300*g* (4 °C), where after the supernatants were removed and the pellets resuspended in 450 µl buffer solution (0.5% BSA in DPBS) and 50 µl Myelin Removing Beads (Miltenyi Biotec, cat#: 130-096-733). The samples were placed at 4 °C for 15 min and a 5 ml buffer solution was added before a 10 min centrifugation at 300*g* (4 °C). The supernatants were discarded and the pellets resuspended in 500 µl buffer solution, and an LS column (Miltenyi Biotec, cat#: 130-042-401) was presoaked in 3 ml buffer solution. The cell suspension was added to the LS column and 2 × 500 µl of buffer solution was added to the column to collect cells remaining in the column. The cell suspensions were kept on ice until sorting, which was performed immediately after the dissociation.

### Fluorescence activated cell sorting

The cell suspensions were analyzed, and 178 Vmat2-Cre;*tdTomato* cells were sorted onto a 384 Smart-Seq2 well plate using a BD FACSMelody and BD FACSChorus software. The particles were initially gated based on the area in forward and side light scatter (FSC-A/SSC-A) to detect cell-sized particles of homogenous character, which were further gated on height and width in two steps (1. SSC-H/SSC-W, 2. FSC-H/FSC-W) to isolate single-cells. To detect *tdTomato* positive cells and exclude single-cell like particles with autofluorescence, the single-cells were gated on the intensity of different settings of the red and far red channels (PE-Cy5(YG)-A, PE-Cy7(YG)-A, (YG)-A) (Figure S6). The cortical sample from the *tdTomato* negative animal was used as a control sample to detect baseline intensity of the red and far red channels (Figure S6A), where after the *tdTomato* intensity threshold could be detected in the (YG)-A channel (Figure S6B). The *tdTomato* positive single-cells were thereafter sorted onto the plate, which was stored at − 80 °C until library sequencing.

### Library sequencing

The single-cell data was generated using the Smart-Seq2 method^20^. To normalize for sequencing depth and gene length, the expression values were computed as reads per kilobase of gene model and million mappable reads (RPKMs). The expression values were thereafter computed per gene^41^ using uniquely aligned reads and correcting for the uniquely alignable positions using MULTo^42^. The samples were first analyzed by demultiplexing the fastq files using deindexer (https://github.com/ws6/deindexer) using the nextera index adapters and the 384 layout. The individual fastq files were thereafter mapped to the relevant genome assembly using the STAR aligner^43^ using 2-pass alignment to improve performance of the de novo splice junction reads, filtered for only uniquely mapping reads.

### Single cell data processing and clustering

The Vmat2-Cre;*tdTomato* cells were sequenced using Smart-Seq2^20^, where a total of 24,582 genes could be detected. The data was analyzed using SCANPY^44^ in Python 3.8.1. The full code and loom file of the unprocessed Vmat2-Cre.*tdTomato* data can be found at: (https://github.com/HannahBanarne/Somatosensory-cortical-Vmat2-analysis). For basic preprocessing, the metrics of general gene expression, ERCC and mitochondrial genes were firstly calculated (SCANPY, queries.mitochondrial_genes(‘mmusculus’), pp.calculate_gc_metrics)^45^. By plotting the distribution of the acquired metrics (Seaborn, jointplot), cells with distributed gene counts and broad gene capture (SCANPY, 'log1p_n_genes_by_counts' > 6.0, 'pct_counts_in_top_50_genes' < 80), high total counts ('log1p_total_counts' > 11), low amount of mitochondrial genes (‘pct_counts_mito’ < 0.1) and less than 10% ERCC were filtered. From the 178 sequenced Vmat2-Cre cells, 158 fulfilled the inclusion criterion and were used for expression analysis. For gene filtering, all genes that were expressed in less than 3 cells in the dataset were excluded, resulting in 18,430 genes that were used for the analysis of the 158 Vmat2-Cre neurons (SCANPY, pp.filter_genes). The counts per cell was thereafter normalized to the median number of counts (SCANPY, pp.normalize_per_cell) followed by normalization (SCANPY, pp.log1p). For data dimension reduction, principal component analysis (PCA) (SCANPY, pp.pca)^46^ was performed using the top 1000 highly variable genes (SCANPY, pp.highly_variable_genes)^47,48^ and the data topology was computed using the UMAP method (SCANPY, pp.neighbors ((n_neighbors = 10, metrics = ‘euclidean’, method = ‘umap’, n_pcs = 9), tl.umap))^35,49^. To investigate if there were any subclusters of Vmat2-Cre neurons with specific gene expression, the cells were clustered into subgroups using the Leiden method^50^ and plotted with UMAP (SCANPY, pl.umap). The clustering resulted in 8 clusters and to detect differences in gene expression among the clusters, the z-scores and adjusted p-values (Benjamini–Hochberg for adjusting the false discovery rate (FDR)) were calculated for the top differentially expressed (DE) genes in each cluster (SCANPY, tl.rank_genes_groups). The gene expression for the top 10 DE genes from each cluster was visualized for all the individual neurons with a heatmap (SCANPY, tl.rank_genes_groups_heatmap) and the mean expression for these genes using a matrix plot (SCANPY, pl.rank_genes_groups_matrixplot). For one of the clusters, the top DE genes included *Fcer1g*, *C1qb*, and *Ctss*, which are marker genes for microglial cells (mousebrain.org), and thus, the 7 cells belonging to this cluster were excluded. The dimension reduction, data topology (n_pcs = 9), clustering and detection of top DE genes were re-performed for the remaining 151 neurons in the dataset.

### Vmat2-Cre marker gene analysis

Analysis of specific gene expression, including *Slc18a2* (*Vmat2*) and marker genes for serotonin synthesis, as well as markers for excitatory and inhibitory neurons was performed for the general Vmat2-Cre population (SCANPY, pl.stacked_violin). To investigate the neurotransmitter input of the Vmat2-Cre population, the ‘targets_and_families.csv’ file was downloaded from Guide to Pharmacology (https://www.guidetopharmacology.org), and the expression prevalence of ligand-gated ion channel subunits and G protein-coupled receptors (GPCRs) were calculated (gene considered expressed if log1p =  > 0.25), where after the top 50 most prevalent genes from the respective receptor type was visualized (SCANPY, pl.stacked_violin).

### Vmat2-Cre cell type prediction

To examine the location and molecular identity of the Vmat2-Cre population, expression of cortical layer marker genes^25^ was examined in the Vmat2-Cre population. To further investigate the cortical identity of the Vmat2-Cre neurons, our dataset was merged and aligned to a subdataset of cortical neurons from the telencephalon projecting neurons dataset published by Zeisel et al. (2018). The ‘l6_r3_telencephalon_projecting_neurons.loom’ dataset was acquired from (http://www.linnarssonlab.org), which contains 28,858 single cells and 27,998 genes obtained by using the 10× Genomics methods. From this dataset, all neurons belonging to cortical pyramidal layers 2/3–6 were isolated for analysis, resulting in 9435 cells and 27,998 genes. These cells were further processed with the same parameters as the Vmat-Cre dataset, except for the exclusion of neurons with high expression of ERCC since the 10 × Genomics method does not include ERCC. The basic preprocessing resulted in 9369 cells and 17,304 genes. To see how well the two datasets aligned with each other, the normalized Vmat2-Cre and Zeisel datasets were concatenated based on all genes from both datasets (SCANPY, concatenate(join = ‘outer’)) and the data dimensions were reduced and visualized with PCA (pp.pca, pl.pca_scatter). Thereafter, the top 5000 highly variable genes (SCANPY, pp.highly_variable_genes) was detected from both datasets, which were further used to concatenate the normalized and scaled datasets of shared gene expression (SCANPY, pp.log1p, pp.scale) and correct the batch effects using matching mutual nearest neighbors (MNN) (SCANPY, external.pp.mnn_correct)^35^. The data dimensions were reduced with PCA (SCANPY, pp.pca) and the data topology visualized with UMAP (pp.neighbors(n_neighbors = 10, n_pcs = 9), pp.umap). The annotated probable cortical locations and clusters estimated by Zeisel et al. were thereafter predicted and assigned to the Vmat2-Cre cells by first training a linear logistic regression with stochastic gradient descent (SGD) training classifier (scikit-learn, SGDClassifier (loss = ‘log’)) on the Zeisel et al. gene expression as predictor variables and the annotated locations or clusters as target labels. The probable location labels were thereafter predicted for the Vmat2-Cre cells and the probability of correct label assignment was computed for each Vmat2-Cre cell (scikit-learn, predict_proba) before mapping back the predicted labels and probability scores to the scaled concatenated dataset and the original Vmat2-Cre dataset. To visualize how well the annotations were predicted for the Vmat2-cells, the probability scores were plotted with a distribution plot (Seaborn, distplot).

### In situ tissue preparation

Two female Vmat2-Cre adult (10 weeks old) mice microinjected with AAVDJ.EF1a-DIO-HTB in the SB1F, were perfused 14 days post injection after sedation in isoflurane (FORANE, Baxter, USA), followed by i.p injection of 0.6 ml (1:1) Ketamin (Ketalar, 10 mg/ml, Pfizer) and Medetomidine (Domitor, 1 mg/ml, Orion Pharma). To minimize the risk of contamination and altered gene expression, the animals were perfused in autoclaved ice-cold 1xPBS. The brains were quickly dissected and in autoclaved ice-cold, 1xPBS, the hemisphere area containing the S1BF was isolated and thereafter embedded in OCT medium (Bio-Optica, Milan, Italy). The tissues were immediately snap-frozen on dry ice in − 80 °C isopentane (Sigma-Aldrich, Germany) and were stored at this temperature until sectioning. The tissues were cryosectioned (Leica Cryocut 1800) into 12 µm slices and collected as series of 6 slides/series onto Superfrost Plus (Thermo Scientific) slides. To prevent mRNA degradation and contamination, the completed series were stored at − 21 °C until sectioning was finished. The slides were stored at − 80 °C until in RNAscope Fluorescent Multiplex kit (Advanced Cell Diagnostics (ACD), cat # 320850) protocol commenced.

### Fluorescent in situ hybridization

The fluorescent in situ hybridization (FISH) was performed using the RNAscope Fluorescent Multiplex kit (Advanced Cell Diagnostics (ACD), cat# 320850) in accordance with ACD guidelines for fresh frozen tissues with minor modifications^51^. In brief: the tissue slides to be used were taken from − 80 °C and immediately fixated in RT 4% PFA in 1 × PBS (Histolab, Sweden) for 15 min before being washed in autoclaved 1 × PBS for 2 min. The tissues were thereafter dehydrated in a step-wise increase of EtOH concentration; 3 min in 50%, 3 min in 70% and 2 times for 5 min in 100% (Merck KGaA, Damstadt, Germany). The slides were placed at RT for 5 min to dry where after a hydrophobic barrier was made around the slide area of interest (2–5 slides/probe and animal) using an ImmeEdge pen (Vector Laboratories, Burlingame, California, USA). The sections were thereafter incubated in Protease IV for 30–40 min at RT followed by 3 times 5 min washing in autoclaved 1xPBS, followed by incubation in target probes (cat #: *Vglut1* (*Slc17a6*): 416631-C2, *Vglut2* (*Slc17a6*): 319171-C2; *Vglut2*: 319171-C3; *Viaat* (*Slc32a1*): 319191-C3; *Vmat2*: 425331-C3; *Rorb*: 444271-C2; *Scnn1a*: 441391-C3, 1:50 in probe diluent cat #: 300041) for 2 h at 40 °C in a hybridization oven (HybEZ II Oven, ACD). The following amplification steps were performed at 40 °C in an oven and the sections were washed 2 times for 2 min in RT washing buffer between each amplification step; AMP 1-FL for 30 min, AMP 2-FL for 15 min, AMP 3-FL for 30 min and AMP 4-FL for 15 min. The coloring step using AMP 4-FL was performed to enable the combination with the viral GFP. Lastly, the slides were washed 2 times 2 min in washing buffer before 30 s incubation in DAPI and mounting in Anti-Fade Fluorescence Mounting Medium (Abcam). The slides were covered with glass slides (Menzel-Gläser) and were left at 4 °C to dry. The slides were stored at this temperature until imaging.

### In situ hybridization image acquisition and quantification

Images of RNAscope treated Vmat2-Cre.HTB SB1F sections were acquired with wide field 20 × magnification using an Olympus BX61WI fluorescence microscope (Olympus, Japan). The channel for each probe was optimized for brightness and contrast during image acquisition and quantification. The RNAscope images were manually quantified using the Fiji (ImageJ 1.52f) Cell Counter plugin^52,53^. All Vmat2-Cre.HTB cells with DAPI overlap were considered cells and one read of the targeted probe could be visualized as one dot. A Vmat2-Cre.HTB cell was considered to be expressing the targeted gene if the overlapping # dots ≥ 3. The result is presented as percentage ± SEM of Vmat2-Cre.HTB cells expressing *Vmat2*, *Vglut1*, *Vglut2*, *Viaat, Rorb and Scnn1a*.

### Immunohistochemistry

One 16-week old male and two 14-week old female Vmat2-Cre;*tdTomato* mice were perfused with PBS followed by 4% FA. The whole brain was dissected out and kept in 4% FA overnight at 4℃. Before slicing, the tissues were mounted in 4% agarose (VWR) and cut into 60 µm thick sections using a vibratome (Leica VT1200). Immunostaining with rabbit anti-GABA antibody (Sigma-Aldrich) 1:750 in blocking solution (5% Donkey Serum (Sigma-Aldrich), 3% BSA (Sigma-Aldrich) in TBS) was performed (10 sections per animal). Cells were stained with DAPI (VWR) and Donkey anti-rabbit 647 antibody (Invitrogen) for visualization. The stained sections were mounted and imaged using an Olympus BX61WI fluorescence microscope (Olympus, Japan) with 10 × magnification. Some images were merged to increase the focus using ImageJ (ImageJ, USA) software. Only tdTomato cells with clear DAPI expression were counted. The result is presented as percentage ± SEM of Vmat2-Cre;*tdTomato* cells expressing GABA.

### Statistics

The electrophysiological data values are presented as mean ± standard error of the mean and the mean values were statistically compared using One-way ANOVA with Bonferroni’s post hoc test (GraphPad Software, Inc., San Diego, CA).

## Supplementary Information

Supplementary Information

## Data Availability

The datasets generated during and/or analysed during the current study are available from the corresponding author on reasonable request.

## References

[1] Woolsey, T. A. & Van der Loos, H. The structural organization of layer IV in the somatosensory region (SI) of mouse cerebral cortex. The description of a cortical field composed of discrete cytoarchitectonic units. *Brain Res.* **17,** 205–242 (1970). 10.1016/0006-8993(70)90079-x 4904874

[2] Cowan AI, Stricker C (2004). Functional connectivity in layer IV local excitatory circuits of rat somatosensory cortex. J. Neurophysiol..

[3] Almási Z, Dávid C, Witte M, Staiger JF (2019). Distribution patterns of three molecularly defined classes of gabaergic neurons across columnar compartments in mouse barrel cortex. Front. Neuroanat..

[4] Binshtok AM, Fleidervish IA, Sprengel R, Gutnick MJ (2006). NMDA receptors in layer 4 spiny stellate cells of the mouse barrel cortex contain the NR2C subunit. J. Neurosci..

[5] Guellmar A, Rudolph J, Bolz J (2009). Structural alterations of spiny stellate cells in the somatosensory cortex in ephrin-A5-deficient mice. J. Comp. Neurol..

[6] Espinosa JS, Wheeler DG, Tsien RW, Luo L (2009). Uncoupling dendrite growth and patterning: single-cell knockout analysis of NMDA receptor 2B. Neuron.

[7] Schubert D, Kötter R, Zilles K, Luhmann HJ, Staiger JF (2003). Cell type-specific circuits of cortical layer IV spiny neurons. J. Neurosci..

[8] Unichenko P (2016). Plasticity-related gene 1 affects mouse barrel cortex function via strengthening of glutamatergic thalamocortical transmission. Cereb. Cortex.

[9] Chu Y-F, Yen C-T, Lee L-J (2013). Neonatal whisker clipping alters behavior, neuronal structure and neural activity in adult rats. Behav. Brain Res..

[10] Qi G, Feldmeyer D (2016). Dendritic target region-specific formation of synapses between excitatory layer 4 neurons and layer 6 pyramidal cells. Cereb. Cortex.

[11] Feldmeyer D (2012). Excitatory neuronal connectivity in the barrel cortex. Front. Neuroanat..

[12] Killackey HP, Sherman SM (2003). Corticothalamic projections from the rat primary somatosensory cortex. J. Neurosci..

[13] Takahashi N, Uhl G (1997). Murine vesicular monoamine transporter 2: Molecular cloning and genomic structure. Brain Res. Mol. Brain Res..

[14] Henry JP (1994). Biochemistry and molecular biology of the vesicular monoamine transporter from chromaffin granules. J. Exp. Biol..

[15] Lebrand C (1996). Transient uptake and storage of serotonin in developing thalamic neurons. Neuron.

[16] Lebrand C (1998). Transient developmental expression of monoamine transporters in the rodent forebrain. J. Comp. Neurol..

[17] Vitalis T (1998). Effects of monoamine oxidase A inhibition on barrel formation in the mouse somatosensory cortex: Determination of a sensitive developmental period. J. Comp. Neurol..

[18] Luo X, Persico AM, Lauder JM (2003). Serotonergic regulation of somatosensory cortical development: Lessons from genetic mouse models. Dev. Neurosci..

[19] Staiger JF (2004). Functional diversity of layer IV spiny neurons in rat somatosensory cortex: quantitative morphology of electrophysiologically characterized and biocytin labeled cells. Cereb. Cortex.

[20] Picelli S (2013). Smart-seq2 for sensitive full-length transcriptome profiling in single cells. Nat. Methods.

[21] Gentet LJ (2012). Unique functional properties of somatostatin-expressing GABAergic neurons in mouse barrel cortex. Nat. Neurosci..

[22] Ren JQ, Aika Y, Heizmann CW, Kosaka T (1992). Quantitative analysis of neurons and glial cells in the rat somatosensory cortex, with special reference to GABAergic neurons and parvalbumin-containing neurons. Exp. Brain Res..

[23] Karagiannis A (2009). Classification of NPY-expressing neocortical interneurons. J. Neurosci..

[24] Kawaguchi Y, Kondo S (2002). Parvalbumin, somatostatin and cholecystokinin as chemical markers for specific GABAergic interneuron types in the rat frontal cortex. J. Neurocytol..

[25] Zeisel A (2018). Molecular architecture of the mouse nervous system. Cell.

[26] Harris JA (2014). Anatomical characterization of Cre driver mice for neural circuit mapping and manipulation. Front. Neural Circuits.

[27] Miceli S (2013). High serotonin levels during brain development alter the structural input-output connectivity of neural networks in the rat somatosensory layer IV. Front. Cell Neurosci..

[28] Lombaert N (2018). 5-HTR2A and 5-HTR3A but not 5-HTR1A antagonism impairs the cross-modal reactivation of deprived visual cortex in adulthood. Mol. Brain.

[29] Staiger JF, Zuschratter W, Luhmann HJ, Schubert D (2009). Local circuits targeting parvalbumin-containing interneurons in layer IV of rat barrel cortex. Brain Struct. Funct..

[30] Conesa A (2016). A survey of best practices for RNA-seq data analysis. Genome Biol..

[31] Scala F (2019). Layer 4 of mouse neocortex differs in cell types and circuit organization between sensory areas. Nat. Commun..

[32] Schaeren-Wiemers N, André E, Kapfhammer JP, Becker-André M (1997). The expression pattern of the orphan nuclear receptor RORbeta in the developing and adult rat nervous system suggests a role in the processing of sensory information and in circadian rhythm. Eur. J. Neurosci..

[33] Jabaudon D, Shnider SJ, Tischfield DJ, Galazo MJ, Macklis JD (2012). RORβ induces barrel-like neuronal clusters in the developing neocortex. Cereb. Cortex.

[34] Ziegenhain C (2017). Comparative analysis of single-cell RNA sequencing methods. Mol. Cell.

[35] Haghverdi L, Lun ATL, Morgan MD, Marioni JC (2018). Batch effects in single-cell RNA-sequencing data are corrected by matching mutual nearest neighbors. Nat. Biotechnol..

[36] Ritchie ME (2015). limma powers differential expression analyses for RNA-sequencing and microarray studies. Nucleic Acids Res..

[37] Johnson WE, Li C, Rabinovic A (2007). Adjusting batch effects in microarray expression data using empirical Bayes methods. Biostatistics.

[38] Franklin, K.B.J, & Paxinos, G. *The Mouse Brain in Stereotaxic Coordinates*, 3rd edition (Academic Press, 2008).

[39] Watson, C., Paxinos, G, & Kayalioglu, G. *The Spinal Cord: A Christopher and Dana Reeve Foundation Text and Atlas*. 1^st^ edition (Academic Press, 2009).

[40] Freitag FB, Ahemaiti A, Jakobsson JET, Weman HM, Lagerström MC (2020). Spinal gastrin releasing peptide receptor expressing interneurons are controlled by local phasic and tonic inhibition. Sci. Rep..

[41] Ramsköld D, Wang ET, Burge CB, Sandberg R (2009). An abundance of ubiquitously expressed genes revealed by tissue transcriptome sequence data. PLoS Comput. Biol..

[42] Storvall H, Ramsköld D, Sandberg R (2013). Efficient and comprehensive representation of uniqueness for next-generation sequencing by minimum unique length analyses. PLoS ONE.

[43] Dobin A (2013). STAR: Ultrafast universal RNA-seq aligner. Bioinformatics.

[44] Wolf FA, Angerer P, Theis FJ (2018). SCANPY: Large-scale single-cell gene expression data analysis. Genome Biol..

[45] McCarthy DJ, Campbell KR, Lun ATL, Wills QF (2017). Scater: pre-processing, quality control, normalization and visualization of single-cell RNA-seq data in R. Bioinformatics.

[46] Pedregosa, F. *et al.* Scikit-learn: Machine Learning in Python. *arXiv* (2012).

[47] Satija R, Farrell JA, Gennert D, Schier AF, Regev A (2015). Spatial reconstruction of single-cell gene expression data. Nat. Biotechnol..

[48] Zheng GXY (2017). Massively parallel digital transcriptional profiling of single cells. Nat. Commun..

[49] McInnes L, Healy J (2018). & Melville, J.

[50] Traag VA, Waltman L, van Eck NJ (2019). From Louvain to Leiden: Guaranteeing well-connected communities. Sci. Rep..

[51] Wang F (2012). RNAscope: A novel in situ RNA analysis platform for formalin-fixed, paraffin-embedded tissues. J Mol Diagn.

[52] Schindelin J (2012). Fiji: an open-source platform for biological-image analysis. Nat. Methods.

[53] Schneider CA, Rasband WS, Eliceiri KW (2012). NIH Image to ImageJ: 25 years of image analysis. Nat. Methods.

[54] Longair MH, Baker DA, Armstrong JD (2020). Simple Neurite Tracer: open source software for reconstruction, visualization and analysis of neuronal processes. Bioinformatics.

[55] Reuden CT, Schindelin J, Hiner MC, DeZonia BE, Walter AE, Arena ET, Eliceiri KW (2020). Image J2: ImageJ for the next generation of scientific image data. BMC Bioinf..

