# Supplementary material for: Targeting barrel field spiny stellate cells using a vesicular monoaminergic transporter 2-Cre mouse line
Source: Sci Rep. 2021 Feb 5;11:3239. doi: 10.1038/s41598-021-82649-8 (PMC7864935; doi:10.1038/s41598-021-82649-8)
Supplement: Supplementary file 1 — Supplementary Information [file 41598_2021_82649_MOESM1_ESM.docx]

**Supplemental information:**

**Targeting barrel field spiny stellate cells using a vesicular monoaminergic transporter 2-Cre mouse line**

Fabio B. Freitag^1^, Aikeremu Ahemaiti#^1^, Hannah M. Weman#^1^, Katharina Ambroz^1^ and Malin C. Lagerström^1^

^1^Department of Neuroscience, Uppsala University, 751 24 Uppsala, Sweden

# shared

Correspondence to Malin.Lagerstrom@neuro.uu.se

**
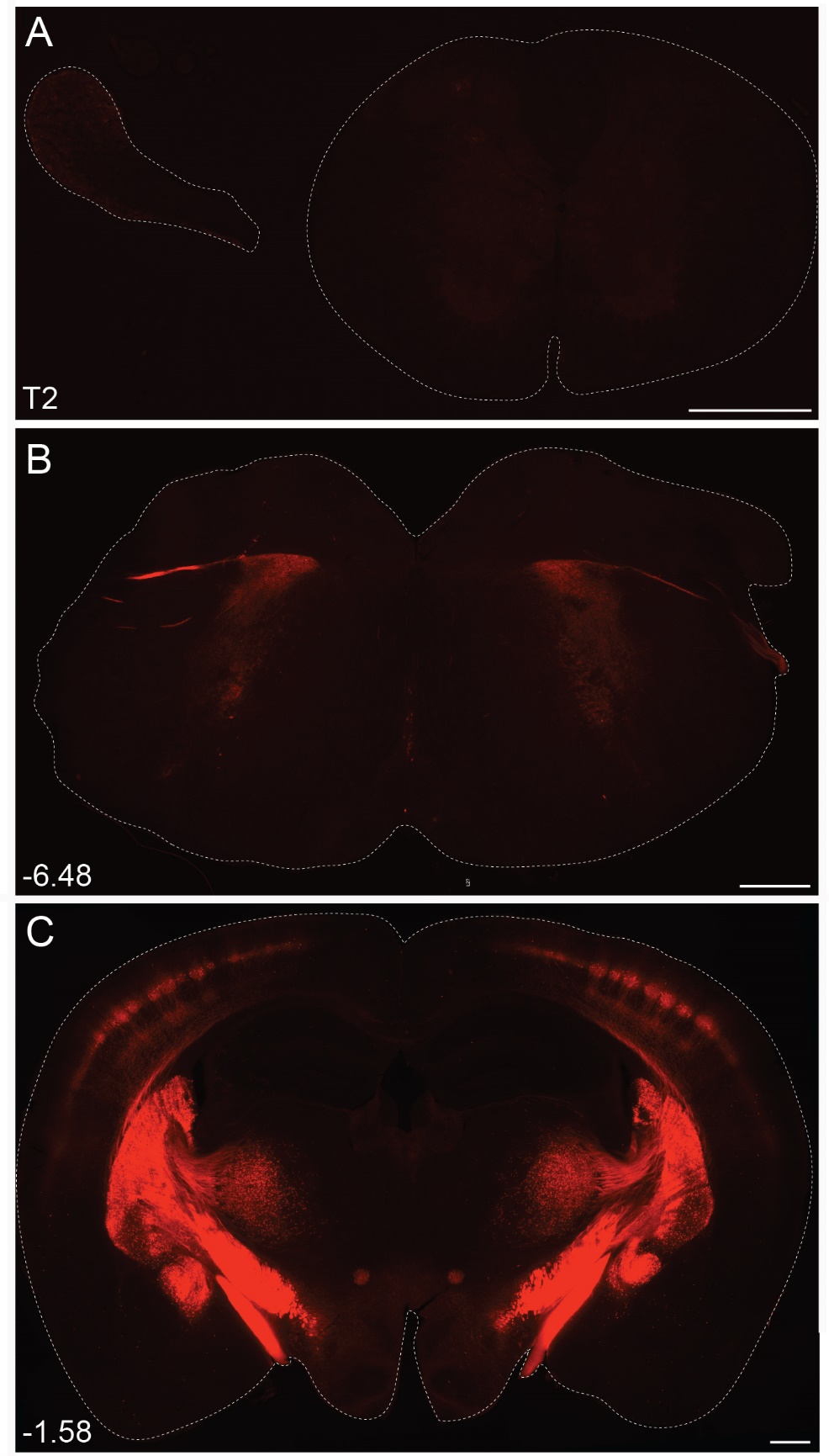
**

**Supplementary figure S1. Vmat2-Cre activity can be detected in thalamo-cortical circuits.** Detection of Vmat2-Cre activity using the reporter line *tdTomato*. Adult coronal sections of mouse neuronal tissue (brain, spinal cord and dorsal root ganglia). **(A)** Weak Vmat2-Cre activity could be observed in the dorsal root ganglia (to the left) and dorsal horn of the spinal cord (to the right) (n=2-3 mice). **(B)** No Vmat2-Cre activity could be detected in the trigeminal nuclei (n=3 mice). **(C)** Strong Vmat2-Cre activity was observed in striatum, thalamus and cortex (n=3 mice). Scale bar: 500 µm. The images were generated using Image J (Image J 1.53e), https://imagej.nih.gov/ij/, and are composites to enable a high resolution.

**Supplementary figure S2. The Vmat2-Cre line displays active Cre expression in S1 barrel field adult mice. (A)** mCherry expression in cortical layer IV neurons from AAV8.hsyn-DIO-mCherry virus injected mice reveals that Cre is still expressed in the barrel field cortex of Vmat2-Cre adult animals. Scale bar, 300µm. **(B)** A Vmat2-Cre neuron expressing mCherry and showing a symmetric dendritic organization (arrow head). Scale bar, 50 µm. **(C-D)** A few (0.5±0.5% per section) Vmat2-Cre neurons with apical dendrites could also be found (arrows). Scale bar, C: 300 µm, D: 50 µm. The images were generated using Fiji (ImageJ 1.52f), https://imagej.net/Welcome.


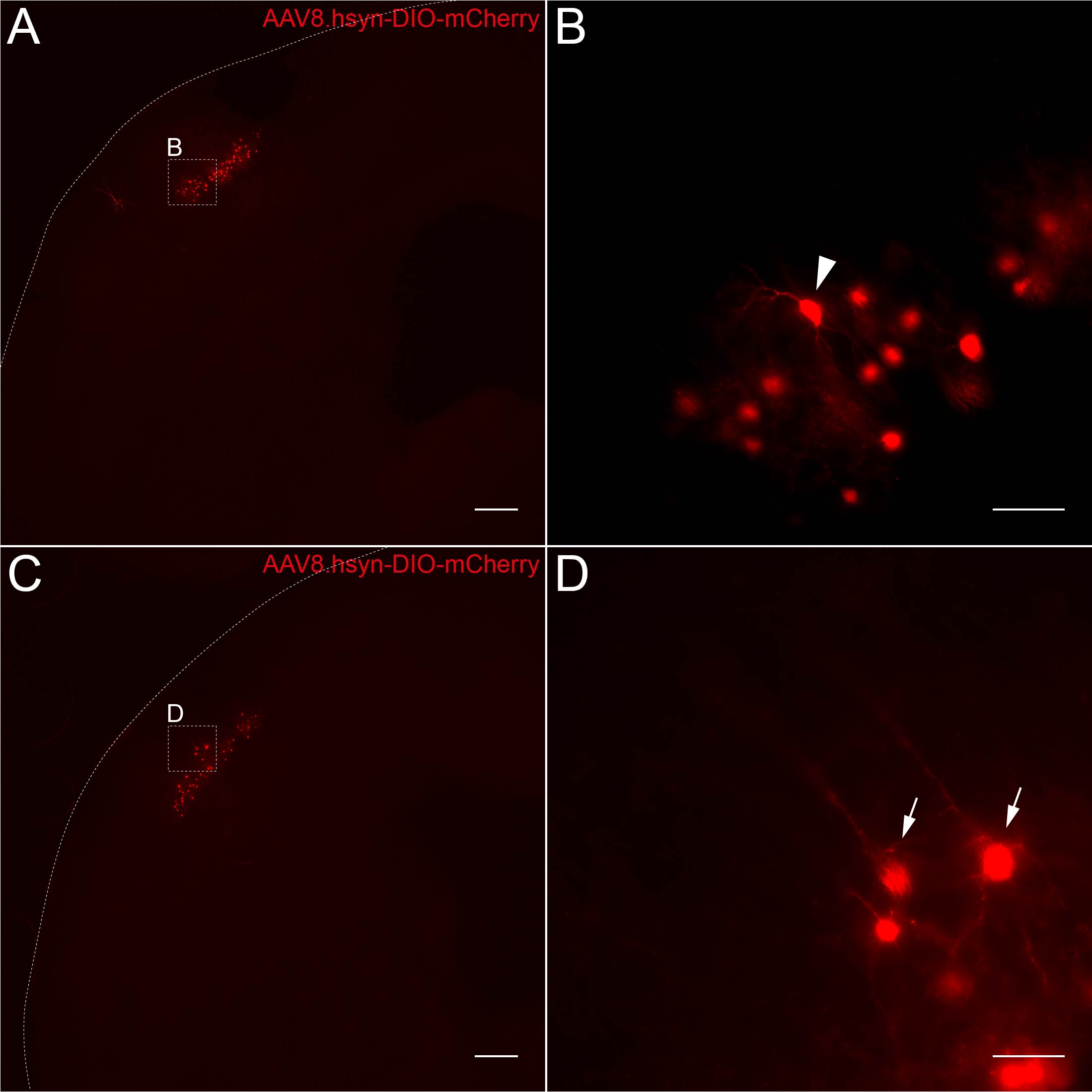


**
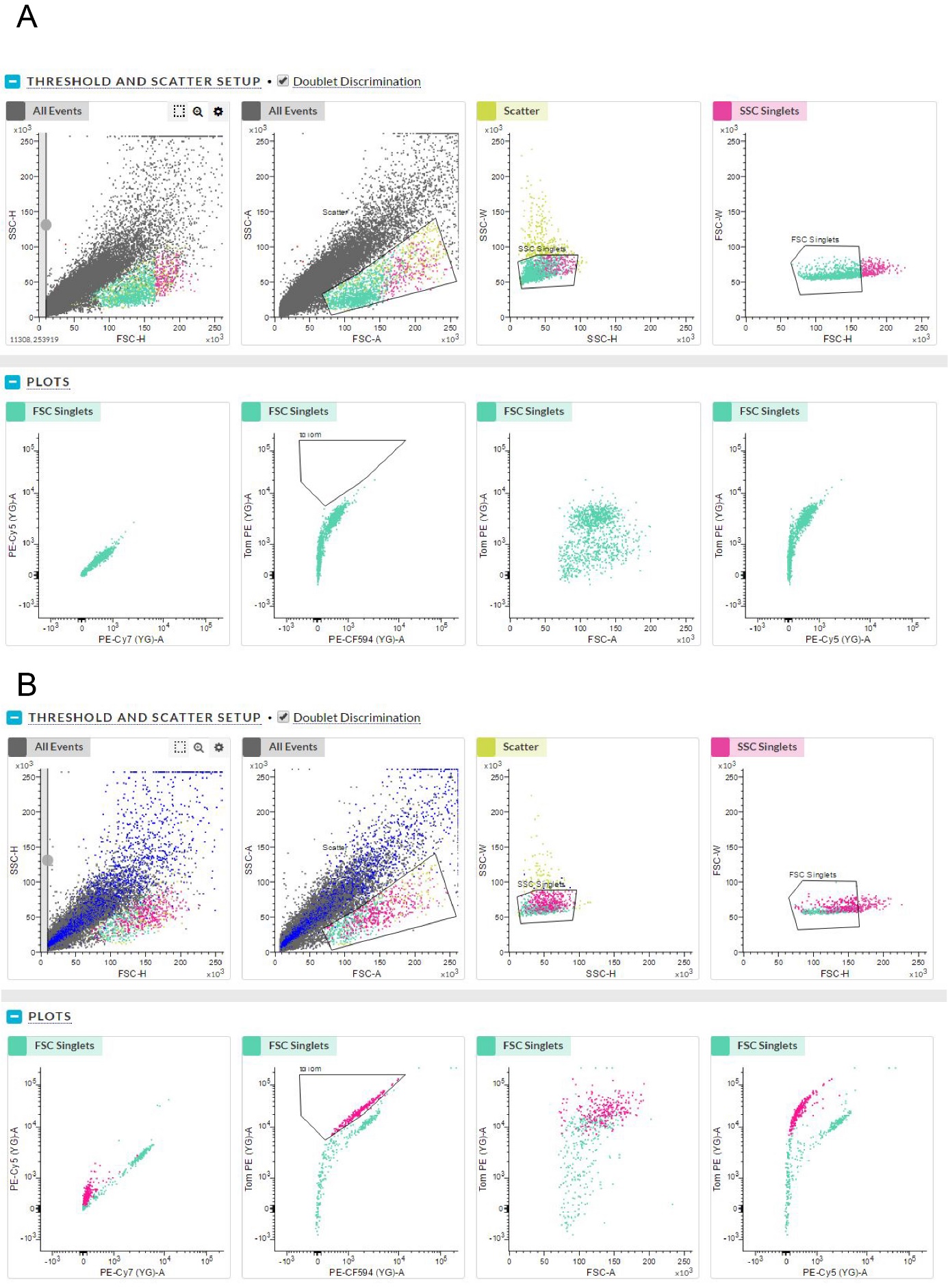
**

**Supplementary figure S3. Fluorescent-activated cell sorting of the Vmat2-Cre;*tdTomato* population.** After tissue dissociation protocol, the particles were first gated based on forward and sideward scatters in a number of steps to isolate particles of single-cell characteristics (top graph rows in fig **A** and **B**). These single-cell like particles were thereafter gated based on intensity of red and far red channels (bottom graphs in fig **A** and **B**) to detect the Vmat2-Cre;*tdTomato* population. A clear red population can be seen in *tdTomato* sample **(B)** that was not detected in the *tdTomato* negative sample **(A)**. The red population in sample **(B)** was sorted onto plate for Smartseq2 sequencing. The images were generated using BD FACSChorusTM, version 1.0, https://www.bdbiosciences.com/en-us/instruments/research-instruments/research-software/flow-cytometry-acquisition/facschorus-software.

**
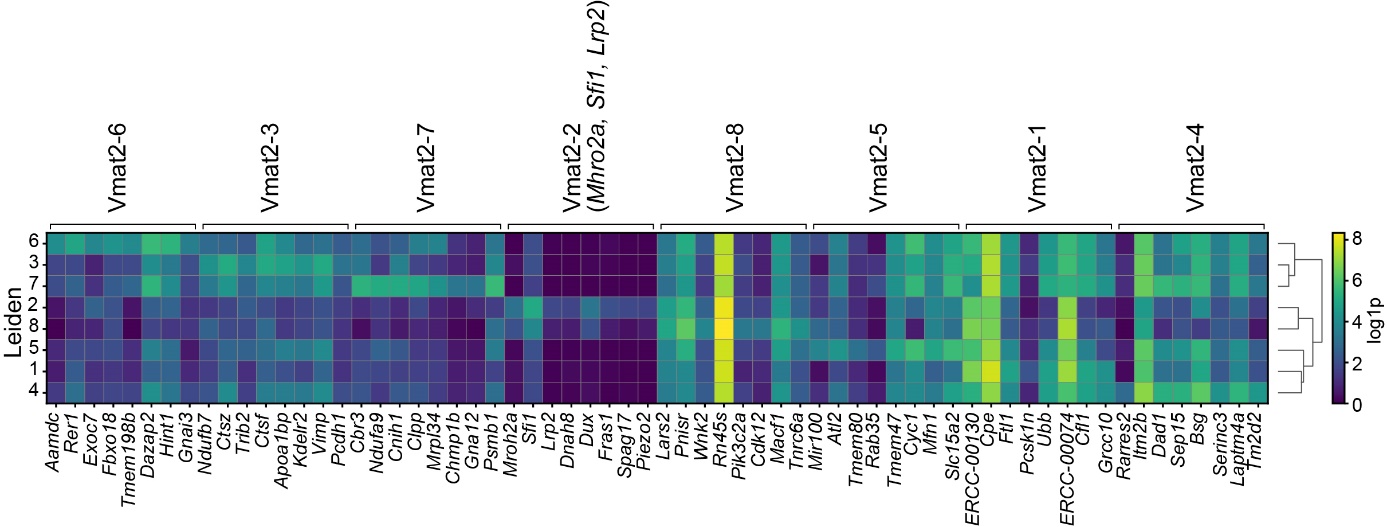
**

**Supplementary figure S4. The Vmat2-Cre;*tdTomato* population is molecularly homogenous on cluster level.** Matrix plot of the mean expression of top DE genes in the Leiden Vmat2-Cre clusters. Significant expression of differentially expressed genes (FDR < 0.05) could only be detected in the Vmat2-2 cluster and therefore, targeted gene analysis was subsequently performed on the whole Vmat2-Cre;*tdTomato* dataset. The image was generated using SCANPY, https://scanpy.readthedocs.io/en/stable/.


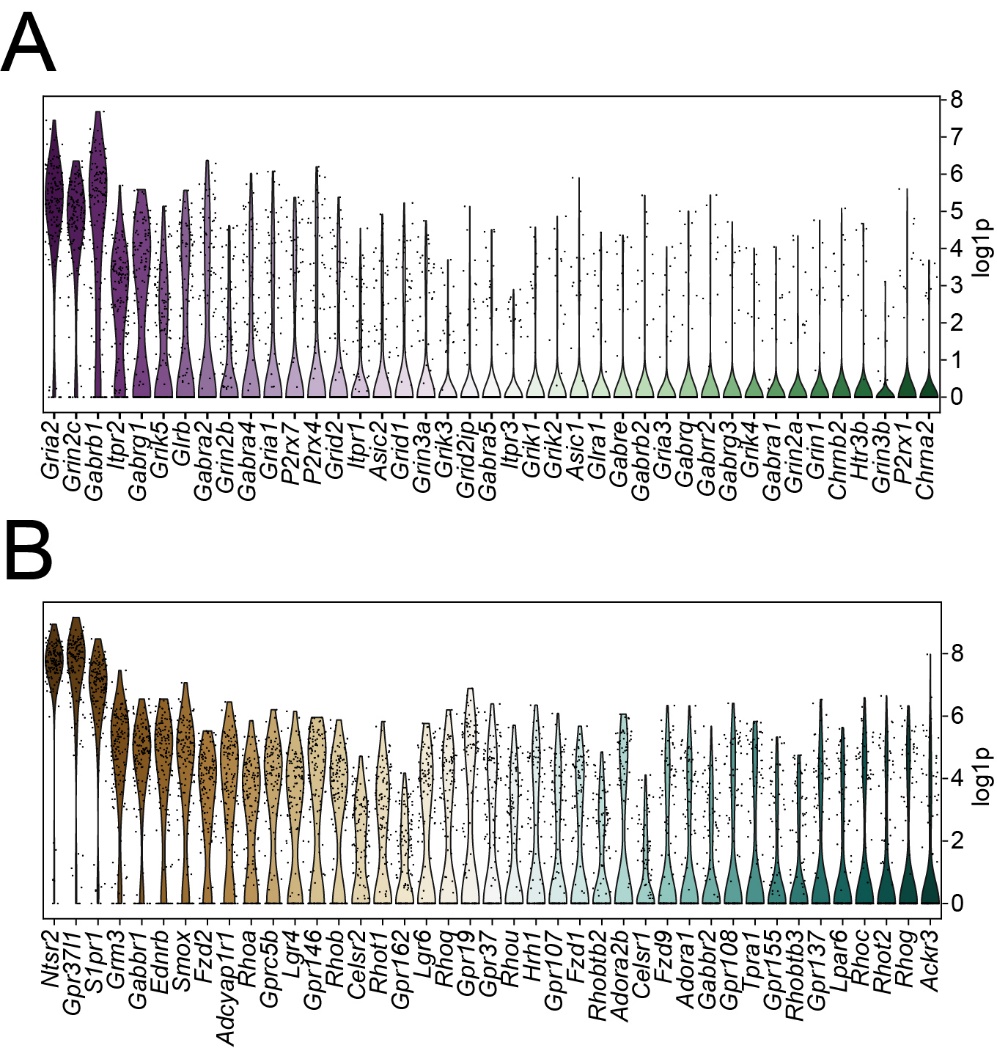


**Supplementary figure S5. The Vmat2-Cre;tdTomato population expresses genes important for glutamatergic, GABAergic and neuropeptide response.** To explore the synaptic input of the Vmat2-Cre*;tdTomato* population, the expression level and prevalence of receptor genes was examined (gene considered prevalent if log1p ≥ 0.25). **(A)** For genes encoding ligand-gated ion channels (Lgics), genes responding to glutamatergic input was mostly prevalent (*Gria2*: 93.38 %, *Grin2c*: 93.38%, *Grik5*: 54.3 %), followed by GABA-responding genes (*Gabrb1*: 86.09 %, *Gabrg1*: 66.89 %). Other Lgic genes expressed by the majority of Vmat2-Cre*;tdTomato* cells were *Glrb* (glycine receptor subunit b) and *Itpr2* (inositol 1,4,5-trisphosphate receptor type 2). **(B)** The most prevalently expressed GPCRs included *Ntsr2* (neurotensin receptor 2), *S1pr1* (sphingosine-1-phosphate receptor 1) and *Ednrb* (endothelin receptor) (*Ntsr2*: 98.7 %, *S1pr1*: 98.0 %, *Ednrb*: 84.8 %). The *Hrh1* (histamine receptor 1), *Gpr107* (neuronostatin receptor) and *Adora* genes (adenosine receptors) were also highly prevalent (*Hrh1*: 47.7 %, *Gpr107*: 46.4 %, *Adora2b*: 41.1 %, *Adora1*: 35.8%). The Vmat2-Cre*;tdTomato* population also showed prevalent expression of the glutamate and GABA metabotropic receptor genes (*Grm3: 93.4 %*, *Gabbr1: 87.4 %, Gabbr2: 35.8 %*). The images were generated using SCANPY, https://scanpy.readthedocs.io/en/stable/.


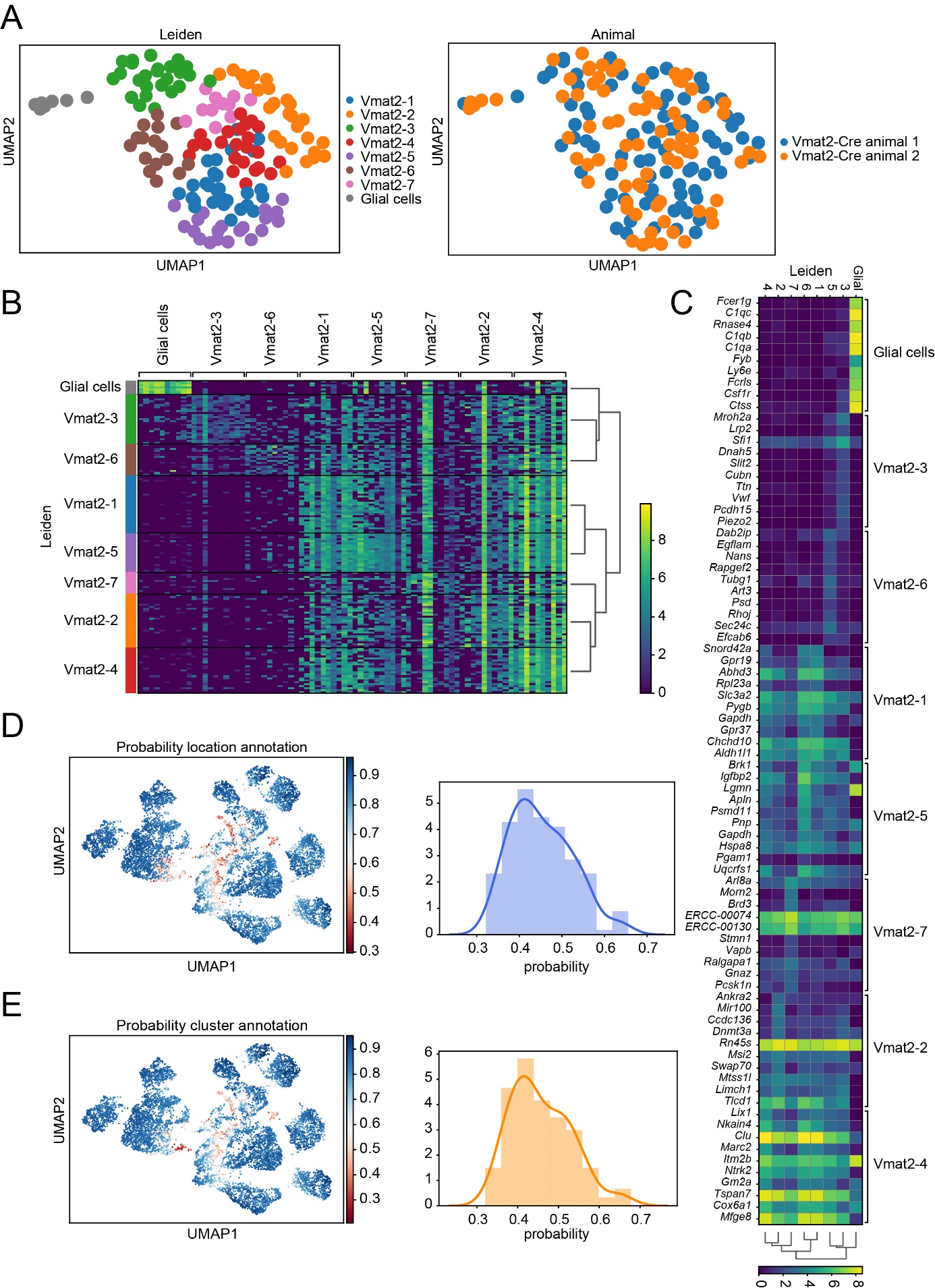


**Supplementary figure S6. Initial analysis of the single-cell mRNA sequenced Vmat2-Cre;*tdTomato* population and validation of Zeisel et al annotated location and cluster assignment of the Vmat2-Cre population. (A-C)** Initial expression analysis of the Vmat2-Cre population revealed that a smaller number of cells expressing glial markers were sequenced. These cells were not included for further analysis. **(A)** Uniform Manifold Approximation and Projection (UMAP) visualization of the Vmat2-Cre neurons according to Leiden clustering and animal origin. **(B)** Heatmap representation of expression of Leiden clustering top differentially expressed (DE) genes in individual Vmat2-Cre neurons. **(C)** The mean expression of top DE genes in the Vmat2-Cre Leiden clusters. **(D-E)** To molecularly investigate the cortical layer location and cluster belonging of the Vmat2-Cre cells, the Vmat2-Cre dataset was mapped to the Zeisel et al (2018) excitatory cortical dataset. **(D)** UMAP visualization of the probability scores of the Zeisel et al annotated location calculated for the assigned Vmat2-Cre population (left) and the distribution of probability scores of correct assignment in the Vmat2-Cre population (right). **(E)** Visualization of the probability scores calculated for the Zeisel et al annotated clusters assigned to the Vmat2-Cre population (left) and the distribution of the probability scores for correct cluster assignment in the Vmat2-Cre population (right). The images were generated using SCANPY, https://scanpy.readthedocs.io/en/stable/.

**Supplementary table S1**. Top 20 differentially expressed genes in cluster Vmat2-2.

| Gene | z-score | FDR |
| --- | --- | --- |
| *Mroh2a* | 8.36 | 6.96E-07 |
| *Sf11* | 7.95 | 2.55E-06 |
| *Lrp2* | 7.3 | 6.12E-05 |
| *Dnah8* | 7.29 | 6,.12E-05 |
| *Dux* | 7.27 | 4.31E-05 |
| *Fras1* | 7.03 | 1.40E-04 |
| *Spag17* | 6.70 | 2.98E-04 |
| *Piezo2* | 6.54 | 3.08E-04 |
| *Dnah5* | 6.45 | 2.07E-04 |
| *Cubn* | 6.38 | 2.98E-04 |
| *Csmd2* | 6.10 | 6.21E-04 |
| *Pkhd1l1* | 5.95 | 1.27E-03 |
| *Zan* | 5.92 | 8.22E-04 |
| *Ryr3* | 5.86 | 7.39E-04 |
| *Pcnxl2* | 5.81 | 1.74E-03 |
| *Hmcn1* | 5.77 | 1.27E-03 |
| *Klc2* | 5.64 | 1.27E-03 |
| *Hydin* | 5.58 | 1.16E-03 |
| *Stab2* | 5.58 | 1.65E-03 |
| *Csmd3* | 5.51 | 1.47E-03 |
